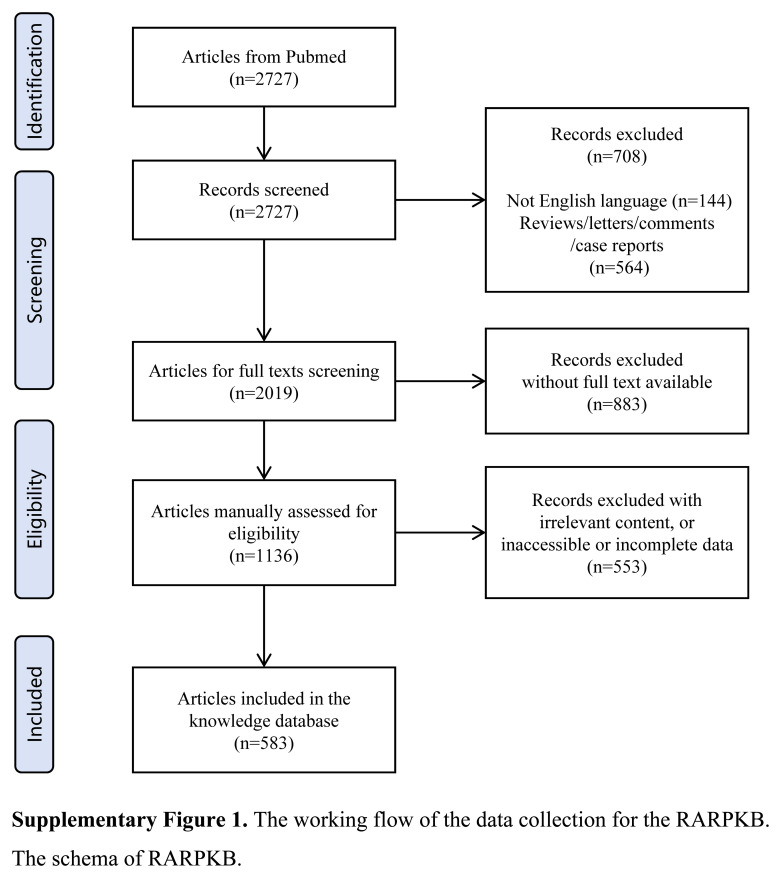# RARPKB: a knowledge-guide decision support platform for personalized robot-assisted surgery in prostate cancer

**DOI:** 10.1097/JS9.0000000000001290

**Published:** 2024-03-18

**Authors:** Jiakun Li, Tong Tang, Erman Wu, Jing Zhao, Hui Zong, Rongrong Wu, Weizhe Feng, Ke Zhang, Dongyue Wang, Yawen Qin, Zheng Shen, Yi Qin, Shumin Ren, Chaoying Zhan, Lu Yang, Qiang Wei, Bairong Shen

**Affiliations:** a Department of Urology, West China Hospital, Sichuan University; b Department of Ophthalmology, West China Hospital, Sichuan University; c Institutes for Systems Genetics, Frontiers Science Center for Disease-related Molecular Network, West China Hospital, Sichuan University; d Chengdu Aixam Medical Technology Co. Ltd, Chengdu; e Department of Computer Science and Information Technologies, Elviña Campus, University of A Coruña, A Coruña, Spain; f Clinical Medical College, Southwest Medical University, Luzhou, Sichuan Province; g Soochow University, Suzhou, China

**Keywords:** knowledge base, prostate cancer, robot-assisted radical prostatectomy, personalized surgery, clinical decision support

## Abstract

**Background:**

Robot-assisted radical prostatectomy (RARP) has emerged as a pivotal surgical intervention for the treatment of prostate cancer (PCa). However, the complexity of clinical cases, heterogeneity of PCa, and limitations in physician expertise pose challenges to rational decision-making in RARP. To address these challenges, the authors aimed to organize the knowledge of previously complex cohorts and establish an online platform named the RARP knowledge base (RARPKB) to provide reference evidence for personalized treatment plans.

**Materials and methods:**

PubMed searches over the past two decades were conducted to identify publications describing RARP. The authors collected, classified, and structured surgical details, patient information, surgical data, and various statistical results from the literature. A knowledge-guided decision-support tool was established using MySQL, DataTable, ECharts, and JavaScript. ChatGPT-4 and two assessment scales were used to validate and compare the platform.

**Results:**

The platform comprised 583 studies, 1589 cohorts, 1 911 968 patients, and 11 986 records, resulting in 54 834 data entries. The knowledge-guided decision support tool provide personalized surgical plan recommendations and potential complications on the basis of patients’ baseline and surgical information. Compared with ChatGPT-4, RARPKB outperformed in authenticity (100% vs. 73%), matching (100% vs. 53%), personalized recommendations (100% vs. 20%), matching of patients (100% vs. 0%), and personalized recommendations for complications (100% vs. 20%). Postuse, the average System Usability Scale score was 88.88±15.03, and the Net Promoter Score of RARPKB was 85. The knowledge base is available at: http://rarpkb.bioinf.org.cn.

**Conclusions:**

The authors introduced the pioneering RARPKB, the first knowledge base for robot-assisted surgery, with an emphasis on PCa. RARPKB can assist in personalized and complex surgical planning for PCa to improve its efficacy. RARPKB provides a reference for the future applications of artificial intelligence in clinical practice.

## Graphical abstract

**Figure:**
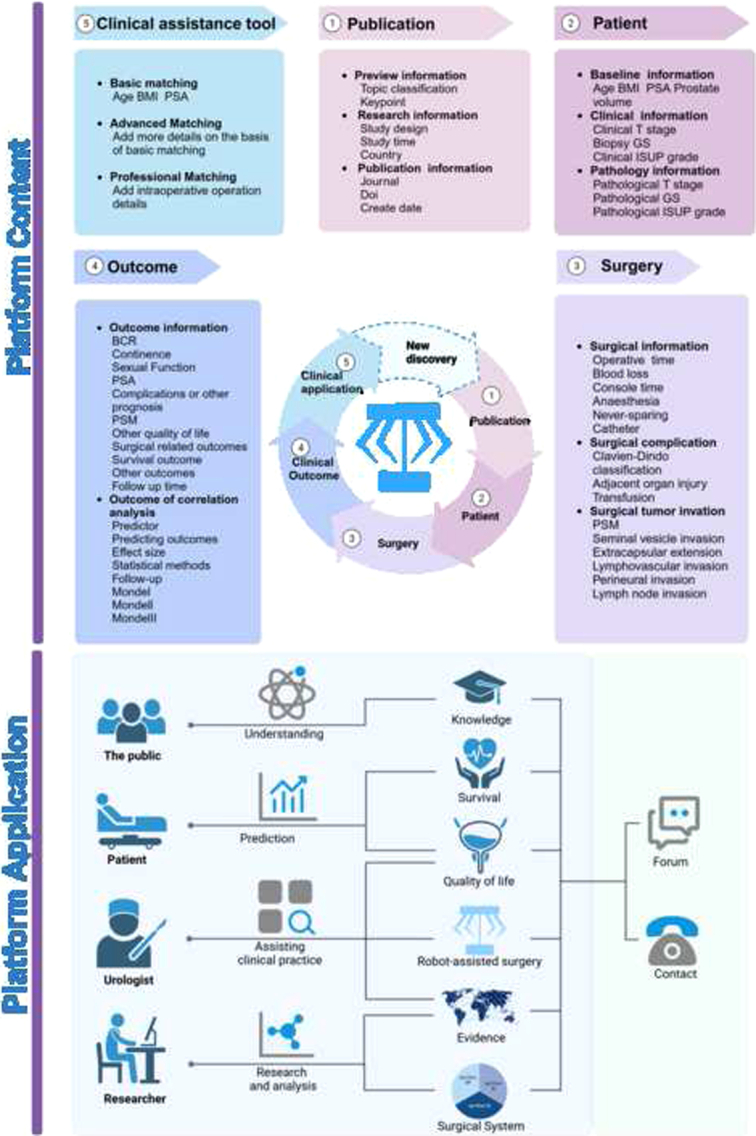

## Introduction

Highlights

- Robot-Assisted Radical Prostatectomy Knowledge Base (RARPKB) is the first specialized knowledge base for robot-assisted surgery. Based on RARP, it is a groundbreaking platform for centralized decision support in complex surgeries.
- Combining extensive research from over two decades with more than 54 000 data entries, RARPKB allows for the input of patient baseline and surgical details to provide personalized, evidence-based decision support. This comprehensive feature enhances outcomes in complex clinical cases of prostate cancer surgery.
- The innovative approach of RARPKB holds potential as a model for future treatment paradigms, extending its applicability to other diseases and medical conditions.

Prostate cancer (PCa) is a common malignancy among men^1^. According to existing research, the early treatment of PCa is of utmost importance^2,3^. In the era of artificial intelligence (AI), robotic surgery has emerged as a crucial approach for PCa treatment^4–6^. Robot-Assisted Radical Prostatectomy (RARP) has superior oncological and functional outcomes^7–10^, and its global adoption has played a crucial role in PCa treatment.

Nevertheless, clinical decision-making regarding RARP has considerable limitations^11,12^. First, the complexity of clinical issues^13^ requires personalized treatment plans tailored to each patient’s condition and preferences^14,15^. Second, the heterogeneity of PCa^16^, both histologically and in various clinical examination results^17,18^, presents challenges as underlying factors yet to be fully explored contribute to a complex and variable disease course^19^. Third, the limitations of clinical experience and decision-making^20,21^ may lead to subjective biases when designing patient treatment plans, resulting in medical errors or suboptimal objective treatment strategies^22^, affecting various aspects of surgical decisions and related patient interventions^23–25^. Any deviations in decision-making can substantially affect the survival and quality of life of patients with PCa and influence the efficiency of medical teams and hospitals. Consequently, addressing these pressing issues has become the central focus of current clinical research.

International institutions advocate the establishment of safer and more scientifically grounded healthcare systems to enhance support and improve medical quality^24,26^. Intelligent systems rely on a foundation of knowledge data, which is crucial for storing and organizing vast amounts of information^27^. Building clinical support systems based on a knowledge database allows data analysis based on scientific evidence and aids medical professionals in making informed decisions regarding disease prevention, screening, diagnosis, treatment, and follow-up^28^. Integrated and networked literature resources offer tremendous convenience and efficiency, enabling users to access information remotely^29^. They also facilitate interdisciplinary research, enabling researchers from various domains to swiftly access and integrate medical information.

Many cohort studies have provided strong evidence for personalized clinical treatment^30^. These studies conducted diverse personalized surveys in different regions, populations, and scenarios, and they have provided critical support to clinical doctors. However, the current universal AI model based on machine learning needs to be more interpretable and trustworthy^31–33^, as it cannot accurately solve personalized clinical RARP problems^34^. In the past, most knowledge bases and standards were universal models (i.e. collective consciousness)^35^. Knowledge bases and models of individual diseases are precise medical models that can provide evidence, acceptance, and trust.

To address this gap, we aimed to synthesize research spanning over two decades and develop the RARP knowledge base (RARPKB). This platform serves as a specialized database concentrating on the complexities of clinical issues, tumor heterogeneity, and limitations of clinical experience in robot-assisted surgery. RARPKB can assist users in making clinical decisions through organized, structured, and evidence-based knowledge, and it has laid the foundation for future AI-driven surgical treatments. Moreover, integration with real data will further promote intelligent medicine.

## Materials and methods

### Literature search

A literature search was conducted in PubMed using “prostatic neoplasms”[Title/Abstract] OR (“prostatic”[Title/Abstract] AND “neoplasms”[Title/Abstract]) OR (“prostate”[Title/Abstract] AND “cancer”[Title/Abstract]) OR “prostate cancer”[Title/Abstract]) AND “robot*“[Title/Abstract] AND 1900/01/01:2021/12/31[Date - Publication].

### Inclusion and exclusion criteria

All publications were searched until December 31, 2021. In total, 2727 articles related to PCa and robotics were screened. Reviews, comments, abstracts, case reports, and articles without full texts; non-English publications; and relevant literature that did not involve robot-assisted PCa surgery or lacked tabular data in the original studies were excluded. (Supplementary Fig. 1, Supplemental Digital Content 2, http://links.lww.com/JS9/C106).

### Platform implementation

**Figure 1:**
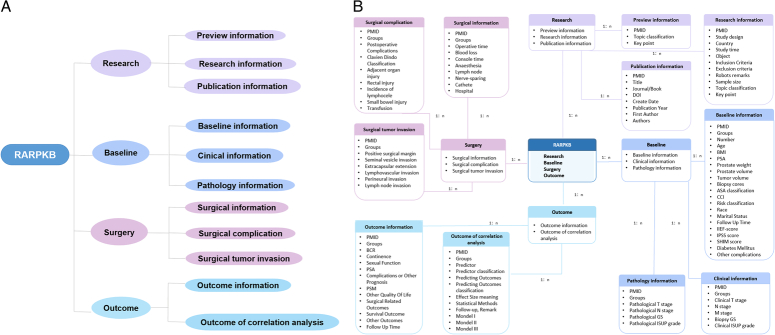
Entity-relationship diagram of the Robot-Assisted Radical Prostatectomy Knowledge Base (RARPKB) structure. (A) Overview of RARPKB. This diagram illustrates the core information of RARPKB and their content overview. (B) This diagram illustrates the data architecture of RARPKB. At the center of the image is the core information of RARPKB, which extends outward with four modules, each containing two to three subinformation tables (pages). The four modules and subinformation tables are represented in four different colors.

After screening the literature, research information was collected and organized, including baseline information, clinical information, pathology information, surgical information, surgical complications, surgical tumor invasion, outcome information, and correlation analysis outcomes. The structure of the database is shown as an entity-relationship diagram in Table 1 and Figure 1.

**Table 1:** Robot-assisted radical prostatectomy knowledge base website page data content table.

Website page	Category	Content
Research	Preview information	PMID, Topic classification, Key point
	Research information	PMID, Study design, Country, Study time, Object, Inclusion Criteria, Exclusion criteria, Robots remarks, Sample size, Topic classification, Key point
	Publication information	PMID, Title, Journal/Book, DOI, Create Date, Publication Year, First Author, Authors
Baseline	Baseline information	PMID, Groups, Number, Age, BMI, PSA, Prostate weight, Prostate volume, Tumor volume, Biopsy cores, ASA physical status classification, CCI, Risk classification, Race, Marital Status, Follow-Up Time, IIEF-score, IPSS score, SHIM score, Diabetes Mellitus, Other complications
	Clinical information of tumor	PMID, Groups, Clinical T stage, N stage, M stage, Biopsy GS, Clinical ISUP grade
	Pathology information of tumor	PMID, Groups, Pathological T stage, Pathological N stage, Pathological GS, Pathological ISUP grade
Surgery	Surgical information	PMID, Groups, Operative time, Blood loss, Console time, Anesthesia, Lymph node, Nerve-sparing, Catheter, Hospital
	Surgical complication	PMID, Groups, Postoperative Complications, Clavien–Dindo Classification, Intraoperative complications (Adjacent organ injury, Rectal injury, Incidence of lymphocele, Small bowel injury), Transfusion
	Surgical tumor invasion	PMID, Groups, PSM, Invasion status (Seminal vesicle invasion, Extracapsular extension, Lymphovascular invasion, Perineural invasion, Lymph node invasion)
Outcome	Outcome information	PMID, Groups, BCR, Continence, Sexual function, PSA, Complications or other prognosis, PSM, Other quality of life, Surgical-related outcomes, Survival outcome, Other outcomes, Follow-up time
	Outcome of correlation analysis	PMID, Groups, General information (Predictor, Predictor classification, Predicting outcomes, Predicting outcomes classification, Effect size meaning, Statistical methods, Follow-up, Remark), Mondel I (Effect size I, 95% CI I, Standard error I, *P* value I, Adjusted I), Mondel II (Effect size II, 95% CI II, *P* value II, Adjusted II), Mondel III (95% CI IIII, *P* value III, Adjusted III)

This table displays the names of all the data contained on the websites’ pages and subpages.

ASA, American Society of Anesthesiologists; BCR, biochemical recurrence; CCI, Charlson comorbidity index; DOI, Digital Object Unique Identifier; GS, Gleason Score; IIEF, International Index of Erectile Function; IPSS, International Prognostic Scoring System; ISUP, International Society of Urological Pathology; PMID, PubMed Unique Identifier; PSA, prostate-specific antigen; PSM, positive surgical margin; SHIM, Sexual Health Inventory for Men.

**Figure 2:**
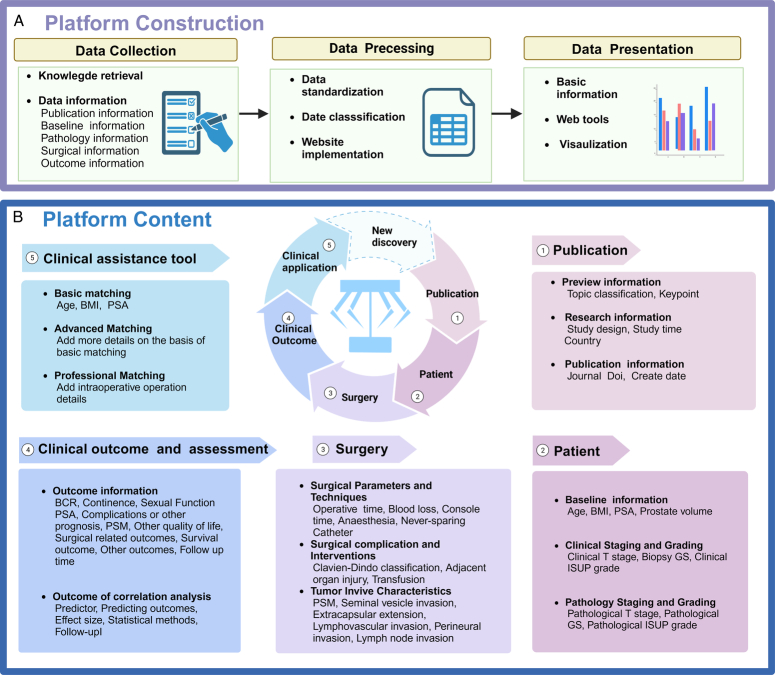
Workflow for the construction of Robot-Assisted Radical Prostatectomy Knowledge Base (RARPKB). The Figure (A) shows the process for constructing the RARPKB platform. They are data collection, data processing, and data presentation, respectively. (B) Illustrates the content of the RARPKB platform, encompassing five main aspects. At its center, a circle represents the cyclic construction process of the platform. For each included study, we collect information on literature, patient demographics, surgical details, clinical outcomes, and outcome assessments. All this information is then integrated and dedicated to clinical application, manifesting as our clinical support tool. As new research is continuously published, we regularly update and supplement the platform, ensuring RARPKB remains an evolving, optimizing, and cyclically constructed network platform. BCR, biochemical recurrence; BMI, body mass index; GS, Gleason score; ISUP, International Society of Urological Pathology; PSA, prostate-specific antigen; PSM, positive surgical margin. Created using BioRender.com.

RARPKB performed data management using MySQL software (version 5.7; Oracle). Web pages were developed using Bootstrap 4.0 (Bootstrap Core Team), and the flask framework was used. Several Java script plugins, such as Datatable (version 1.10.10; SpryMedia Ltd.) and ECharts (version 5.0; Apache), were used to create and visualize the data tables. Figures were created using the online platforms ECharts (https://echarts.apache.org) and BioRender.com (https://app.biorender.com). User feedback surveys were conducted on the Wenjuanxing platform (https://www.wjx.cn; Changsha Ran Xing Science and Technology). The workflow for the RARPKB construction is presented in Figure 2.

### Software for bibliometric analysis

This study used VOSviewer8 (Centre for Science and Technology Studies) as a software tool to perform bibliometric analysis. This tool was used to analyze author collaboration. We also identified the countries with the highest number of publications.

### Validation

We used two approaches to validate the clinical efficacy and utility of the data. First, we configured the information of 15 patients with PCa, including age, body mass index (BMI), prostate-specific antigen (PSA), pathology, and surgery details, into RARPKB and ChatGPT-4. The questions covered surgical recommendations, supporting references, and postoperative complications (Supplemental Digital Content 1, http://links.lww.com/JS9/C105). Following the feedback received from the 15 inquiries, a series of tests were conducted to compare the clinical effectiveness of the assistive features of RARPKB.

Second, we used the System Usability Scale (SUS)^36,37^ and the Net Promoter Score (NPS)^38^ to assess usability and user loyalty of RARPKB. The questionnaire was administered to 20 prospective users, more than 75% of whom were clinicians. We calculated the SUS scores and NPS on the basis of the feedback and scoring algorithms.

### Ethics statement

This study was performed in accordance with the Standards for Quality Improvement Reporting Excellence criteria^39^.

## Results

### Sample

Only articles with complete information were collected to obtain accurate information and raw data, excluding publication type and language (N=1591), leaving 1136 articles. Further screening of the complete texts excluded irrelevant content, or inaccessible or incomplete data (N=553). Ultimately, 583 original studies were included in the knowledge database.

### RARPKB

We developed RARPKB, a knowledge base and web platform dedicated to robot-assisted surgery, specifically RARP. The interface was designed to facilitate users’ effortless browsing, searching, and exploring of the entire database content. All extracted information was standardized and classified. Users can visit the knowledge base at http://rarpkb.bioinf.org.cn for personalized applications in the tool module and communication in the forum module.

### Statistics of the RARPKB platform

A total of 583 research studies were identified during the search, of which 1589 were cohort information and 11 986 were records, generating 54 834 data points. A total of 1 911 968 patients were included in the knowledge base with an average age, BMI, and PSA level of 52.43 years, 26.04 kg/m^2^, and 7.27 ng/mL, respectively.

Surgical information included intraoperative recording time (three items: operative time, console time, and anesthesia duration; 708 records), blood loss (one item; 584 records), lymph node dissection (four items; 335 records), and nerve-sparing procedures (three items; 739 records). The content features of RARPKB are listed in Table 2.

**Table 2:** Robot-assisted radical prostatectomy knowledge base content features.

Item	Number
Publication count	583
Patient	
Sample size	1 911 968
Age (y)	52.43
BMI (kg/m^2^)	26.04
PSA (ng/ml)	7.27
Publication count for surgical information	
Operative time	601
Console time	80
Anesthesia duration	27
Estimated blood loss	584
ePLND	108
Lymph nodes removed	104
Nerve-sparing procedure	302
Unilateral NS	205
Bilateral NS	232
Lymph node count	64
Positive lymph nodes	59
Catheter removal	158
Hospital stay	372
Hospital readmission	34
Clavien–Dindo classification	287
Transfusion	141
Positive surgical margins	304
Seminal vesicle invasion	32
Extracapsular extension	42
Lymphovascular invasion	22
Perineural invasion	35
Lymph node invasion	22
Intraoperative complications	13
Postoperative complications	141
Topic frequency	
AI	2
Anatomic structure	22
BCR	22
Biopsy-related	5
Comparison of surgical techniques	88
Complications	30
Economy	15
Focus on RARP	67
Hospital/surgeon/staff	20
Intraoperative operation	162
Learning curve	19
Local experience	36
Medication	14
MRI	20
New technique	11
Other prognosis	42
Other topic	20
Other treatments	12
Pathology	17
Postoperative treatment	14
Prediction	88
PSM	9
QoL	71
Salvage RARP	13
Sexual function	18
Special populations	28
Supplementary examination	27
Urinary incontinence	45

This table presents the statistics in RARPKB, including the total sample size and baseline mean of the included studies, number of publications on surgical-related information, and number of research topics.

AI, artificial intelligence; BCR, biochemical recurrence; BMI, body mass index; ePLND, expanded pelvic lymph node dissection; NS, nerve-sparing; PSA, prostate-specific antigen; PSM, positive surgical margin; QoL, quality of life; RARP, robot-assisted radical prostatectomy; RARPKB, Robot-Assisted Radical Prostatectomy Knowledge Base.

Knowledge content statistics included word clouds for abstracts, a Sankey diagram for predictive information (a total of 117 corresponding relationships and 696 entries), and various statistical charts for different types of information (six statistical tables covering details of surgical procedures, prognostic outcomes, and research topics). The knowledge content statistics are detailed on the aforementioned website.

**Figure 3:**
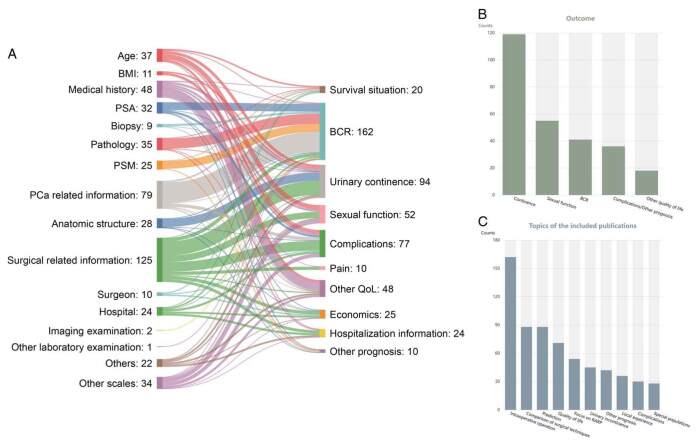
Statistics on the website of Robot-Assisted Radical Prostatectomy Knowledge Base (RARPKB). (A) Sankey diagram of predictive factors correlated with outcomes. A review of the literature has identified several statistically significant predictive factors (*P*<0.05). These are visually represented in a Sankey diagram, illustrating the correlations between these factors and various outcomes. The left side of the diagram lists the predictive factors, while the right side details the potential outcomes. The thickness of the connecting lines corresponds to the number of research papers that discuss each predictive factor and its associated outcomes. (B) Postoperative outcome statistics. This section graphically presents the volume of research literature concerning different postoperative outcomes. The diagram clearly indicates that quality of life, particularly urinary control, is a focal point in the research following robotic prostate cancer surgery. (C) Statistics of topics in the included publications. The diagram displays the distribution of research themes in the included literature. It highlights that the most researched topic is the intricacies of intraoperative procedures (intraoperative operation), followed by comparative studies of robotic versus other surgical techniques (comparison of surgical techniques), predictive research (prediction), studies related to quality of life (quality of life), and research specifically focusing on RARP (focus on RARP). BCR, biochemical recurrence; BMI, body mass index; PSA, prostate-specific antigen; PSM, positive surgical margin.

Additionally, a bibliometric analysis was conducted to provide an overview of the current developments in RARP research. To explore the countries/regions that contributed the most to this field, we analyzed national publication counts. According to the results, the top five countries with the most publications were the United States (131), Japan (75), Italy (66), South Korea (47), and China (44). To further investigate author collaboration, we performed a co-authorship analysis of all publications. It showed that the top five people in the total link strength ranking by weight were Artibani Walter (198), Brunelli Matteo (186), Tafuri Alessandro (186), Amigoni Nelia (161), and Rizzetto Riccardo (161) (Fig. 3).

### Application of the RARPKB platform

#### Knowledge-guide decision support tool

**Figure 4:**
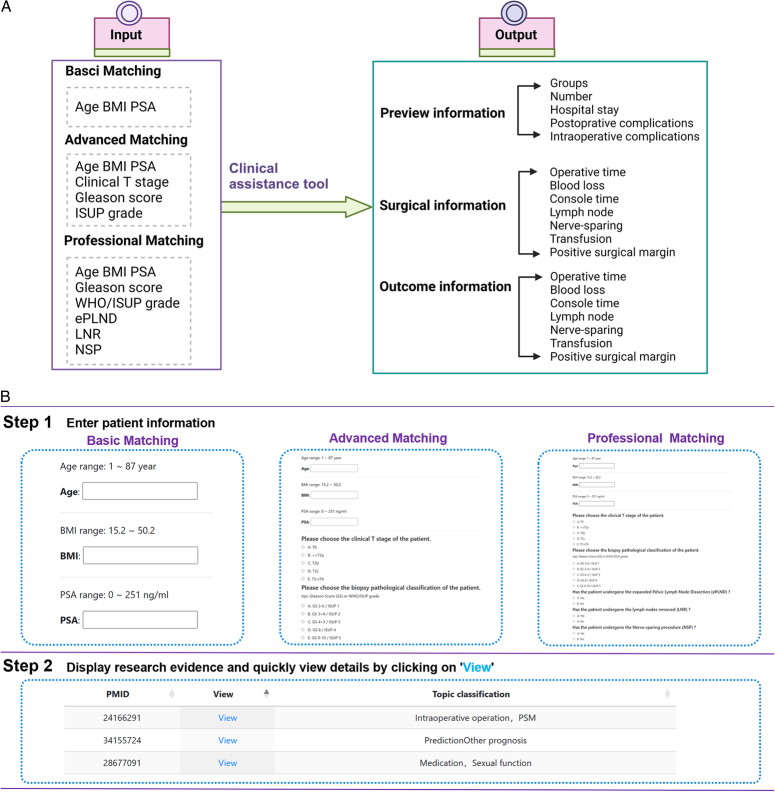
Instruction for the knowledge-clinical decision support tool. The tools page on the Robot-Assisted Radical Prostatectomy Knowledge Base (RARPKB) website allows users to use the platform features. Visual representation the Knowledge-Clinical Decision Support Tool. The diagram shows the specific content of input and output. As in Step 1, users can submit relevant information about the patient. In this step, three modes are achieved on the basis of different requirements: basic, advanced, and professional matching. The information required for each method varied accordingly. Reference evidence can be retrieved after the user submits it, as shown in Step 2. Provide the PMID and topic classification that display matched evidence for users to browse and consider quickly. Click “View” to display the details of the reference evidence. (A) The pipeline of clinical assistant tools. (B) The analytical process within clinical assistant tools. BMI, body mass index; ePLND, expanded pelvic lymph node dissection; ISUP, International Society of Urological Pathology; LNR, lymph nodes removed; NSP, nerve-sparing procedure; PMID, PubMed Unique Identifier; PSA, prostate-specific antigen.

The knowledge-guide decision support tool in RARPKB has three browsing options: basic, advanced, and professional. The tool automatically matches and outputs research evidence by inputting patient information. The resulting page displays the research information, surgical details, outcomes, and prognoses, providing a reference for users (Fig. 4).

#### Basic matching

This module targeted the general public and patients with limited clinical examination results, thereby providing a preliminary impression of the RARP-related research. Basic matching was performed by typing patient age, BMI, and PSA level into a basic matching column and clicking the “Submit” button on the left. All studies in the table were hyperlinked. Pressing the “View” generated a new table view displaying knowledge related to the patient’s condition.

#### Advanced matching

Building upon basic matching, this module allows researchers and patients who have undergone PCa-related examinations to input additional patient classifications and surgical details, further refine the matching results, and make them more targeted. These new entries include the American Society of Anesthesiologists physical status classification, Commodity Channel Index, risk group, race group, and WHO/International Society of Urological Pathologists grade, all of which represent optional information. Additionally, if certain information is unavailable to the patient, the user may leave it blank.

The resulting page provides more specific information. By clicking on the hyperlinks within the table, users can be directed to supplementary tables containing more detailed information on the surgical procedure and prognosis.

#### Professional matching

Designed for professional urology surgeons, this module includes expanded pelvic lymph node dissection, lymph nodes removal, and nerve-sparing procedures. These intraoperative details made the retrieval more accurate and personalized. After entering, the tool outputs research lists containing precise evidence regarding the patients’ perioperative status and postoperative complications for further surgical decision-making.

### Applications

This platform can be used in various applications conducive to personalized treatment and research communities.

**Figure 5:**
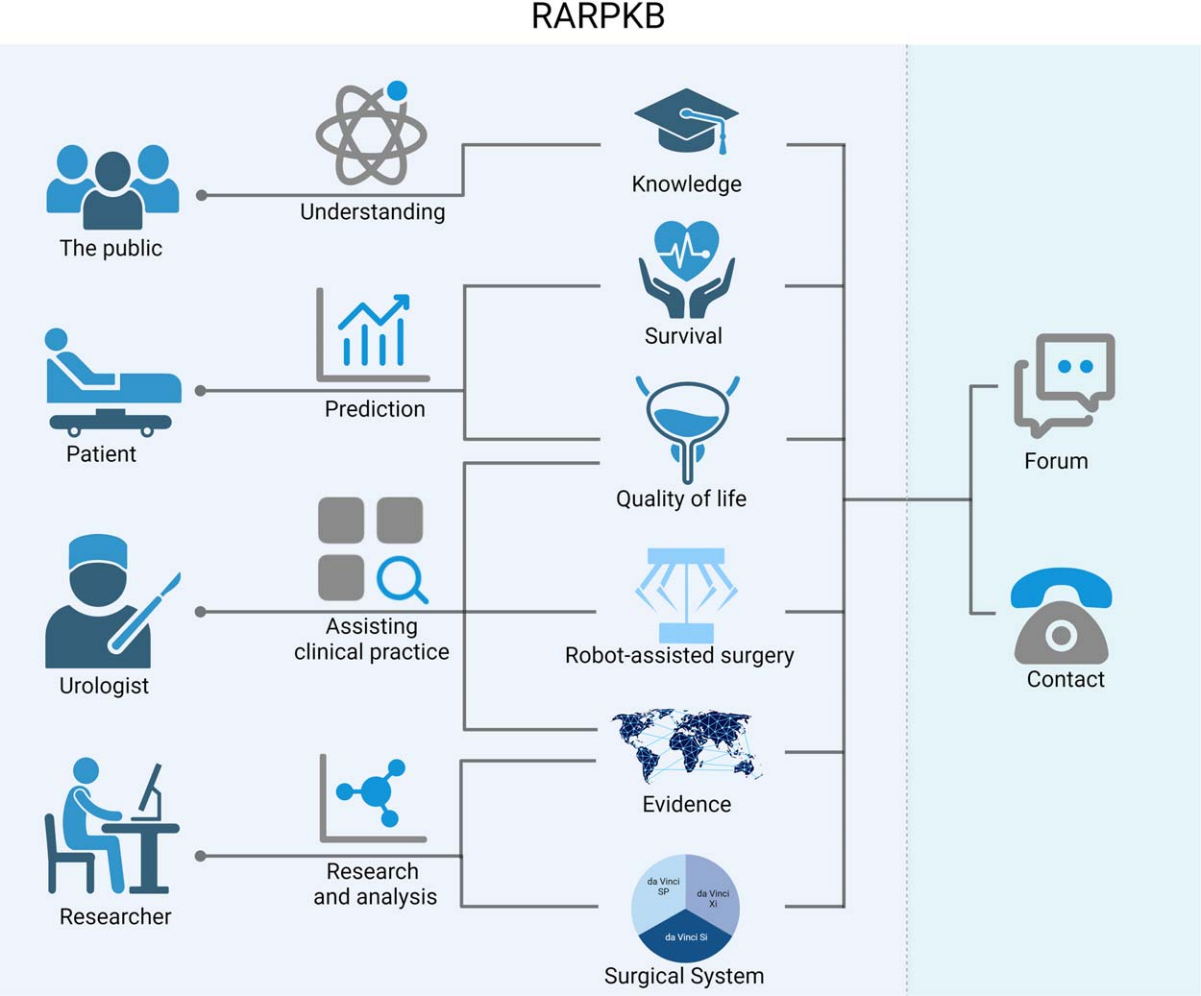
Illustrative function diagram of applications of robot-assisted radical prostatectomy (RARP). Robot-Assisted Radical Prostatectomy Knowledge Base (RARPKB) is applied to a range of user groups, and the image shows the four primary categories of users it pertains to. For the general public, it provides easy access to knowledge regarding RARP for prostate cancer and provides various statistical results. It has clinical applications for both doctors and patients. Patients can easily understand and refer to postoperative prognoses, including survival and quality of life. For doctors, it can facilitate patient surgical plan predictions or references, assist in surgical decision-making, and enable rapid access to RARP-related knowledge. For researchers, it provides quick access to professional expertise. It allows data analysis through RARPKB to meet the needs of different industries, such as clinical and engineering. Finally, all users can engage in accessible communication and feedback. Created using BioRender.com.

1. To our best knowledge, no other database currently contains information on robotic surgery cohorts, including patient data, histopathology, surgical procedures, complications, and prognostic outcomes.
2. This knowledge base has an easy-to-understand interface that inexperienced users can conveniently use.
3. For the general public, users can acquire a fundamental understanding of RARP, its applications, and relevant information regarding its efficacy.
4. For patients, the knowledge-based clinical decision support tool offers valuable information concerning the choice of surgical approach and cancer prognosis. It considers factors such as age, BMI, race, and PSA levels to ensure safety and effectiveness.
5. For urologists, this tool can offer valuable evidence for decision-making based on various factors, for example, patients’ baseline information, nerve-sparing procedures, blood transfusion volume, intraoperative complications, and postoperative complications.
6. For researchers, the RARPKB dataset can be downloaded from the webpage, enabling further analysis, optimization, and enhancement of robot-assisted surgical systems (Fig. 5).

### Validation of the RARPKB platform

We established 15 sets of patient information for basic, advanced, and professional matching on the platform. Compared with ChatGPT-4, RARPKB outperformed in authenticity (100% vs. 73%), matching (100% vs. 53%), personalized recommendations (100% vs. 20%), matching of patients (100% vs. 0%), and personalized recommendations for complications (100% vs. 20%). All outputs were standardized. In addition, ChatGPT-4 uniformly recommended RARP but had a lower focus in its reference publication for RARP (7%). RARPKB, like ChatGPT, included data up to 2021 but highlighted more recent research in outputs (100% vs. 0%) (Table 3, Supplemental Digital Content 1, http://links.lww.com/JS9/C105).

**Table 3:** Comparative assessment of robot-assisted radical prostatectomy knowledge base and ChatGPT-4 test outcomes.

Number	Matching type	Recommended RARP	Authenticity of publications	Matching of publications	Personalized recommendations for publications	Matching of patients	Focus on RARP	Recent research (5 y)	Personalized recommendations for complications	Standardized output results
ChatGPT-4										
1	Basic	**√**	**√**	**√**	×	×	×	×	×	×
2	Basic	**√**	**√**	**√**	×	×	×	×	×	×
3	Basic	**√**	**√**	**√**	×	×	×	×	×	×
4	Basic	√	×	×	×	×	×	×	×	×
5	Basic	√	√	√	√	×	×	×	√	×
6	Advanced	√	√	√	×	×	×	×	×	×
7	Advanced	√	√	√	×	×	×	×	√	×
8	Advanced	√	√	×	×	×	×	×	×	×
9	Advanced	√	√	×	√	×	×	×	√	×
10	Advanced	√	√	×	×	×	×	×	×	×
11	Professional	√	√	√	×	×	×	×	×	×
12	Professional	√	×	×	×	×	×	×	×	×
13	Professional	√	×	×	×	×	×	×	×	×
14	Professional	√	√	√	√	×	√	×	×	×
15	Professional	√	×	×	×	×	×	×	×	×
	√ %	100%	73%	53%	20%	0%	7%	0%	20%	0%
RARPKB										
1	Basic	√	√	√	√	√	√	√	√	√
2	Basic	√	√	√	√	√	√	√	√	√
3	Basic	√	√	√	√	√	√	√	√	√
4	Basic	√	√	√	√	√	√	×	√	√
5	Basic	√	√	√	√	√	√	×	√	√
6	Advanced	√	√	√	√	√	√	√	√	√
7	Advanced	√	√	√	√	√	√	√	√	√
8	Advanced	√	√	√	√	√	√	×	√	√
9	Advanced	√	√	√	√	√	√	√	√	√
10	Advanced	√	√	√	√	√	√	√	√	√
11	Professional	√	√	√	√	√	√	√	√	√
12	Professional	√	√	√	√	√	√	√	√	√
13	Professional	√	√	√	√	√	√	√	√	√
14	Professional	√	√	√	√	√	√	√	√	√
15	Professional	√	√	√	√	√	√	√	√	√
	√ %	100%	100%	100%	100%	100%	100%	80%	100%	100%

This table delineates the nine evaluative dimensions across which the tests were conducted. Fifteen questions were posed, with quintets of these queries corresponding to the basic matching, advanced matching, and professional matching sections on the RARPKB’s Tools page. The table sequentially shows the performance metrics of ChatGPT-4 in the upper section, juxtaposed with the RARPKB’s test outcomes in the lower section. The fulfillment of individual test stipulations is denoted via checkmarks (√) and cross-marks (×).

RARP, robot-assisted radical prostatectomy; RARPKB, Robot-Assisted Radical Prostatectomy Knowledge Base.

**Figure 6:**
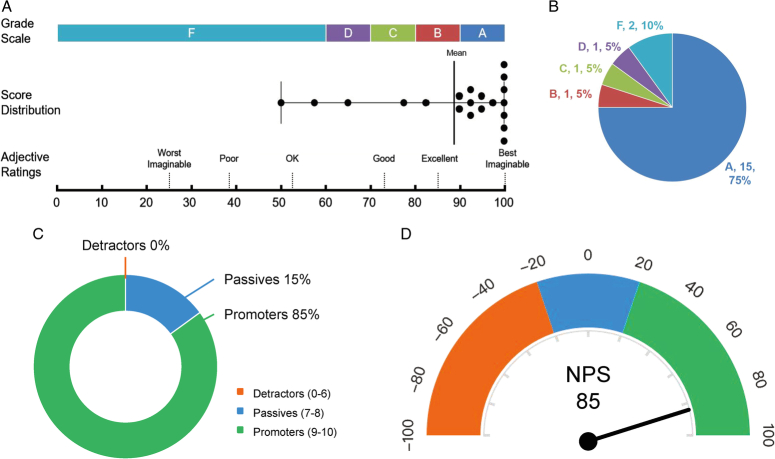
Validation results of the scales for Robot-Assisted Radical Prostatectomy Knowledge Base. The figure outlines the validation outcomes of Robot-Assisted Radical Prostatectomy Knowledge Base. (A) The results for the System Usability Scale are displayed, featuring the grade scale, distribution of scores among 20 test users, and adjective ratings. Each point in the graph represents an individual user’s score, with a mean score of 88.88 delineated by a black line. (B) Grade distribution of the System Usability Scale, in which grade A accounts for 75%. (C) Dispersion of scores on the Net Promoter Score scale. According to the standard criteria of the scale, each user score can be classified into one of three categories. In this study, the percentage of detractors is zero. (D) Calculated Net Promoter Score value on the basis of the standard criteria of 85.

RARPKB usability assessment: we surveyed 20 potential users: clinicians, 75%; researchers, 15%; and public, 10%. Postuse, the average SUS score was 88.88±15.03 (Table 4). According to SUS grading standards, RARPKB rated as “excellent” (Fig. 6a). Grade A samples constituted 75% (Fig. 6b).

**Table 4:** Results of system usability scale and net promoter score scoring metrics

User	SUS score	SUS grade	NPS score	NPS group
1	100	A	10	Promoters
2	92.5	A	10	Promoters
3	100	A	10	Promoters
4	90	A	9	Promoters
5	100	A	10	Promoters
6	82.5	B	10	Promoters
7	90	A	9	Promoters
8	100	A	10	Promoters
9	100	A	10	Promoters
10	92.5	A	10	Promoters
11	50	F	8	Passives
12	95	A	10	Promoters
13	100	A	10	Promoters
14	97.5	A	10	Promoters
15	65	D	8	Passives
16	57.5	F	8	Passives
17	77.5	C	9	Promoters
18	100	A	10	Promoters
19	92.5	A	10	Promoters
20	95	A	9	Promoters
All	88.88±15.03		85	

This table shows the individual-scale outcomes of the users. Additionally, it shows the mean SUS score alongside the NPS outcomes.

NPS, net promoter score; SUS, system usability scale.

RARPKB NPS: the average NPS value was 9.5±0.76 (10). Among users, 85% were promoters (score, 9–10), 15% were passives (score, 7–8), and 0% were detractors (score, 0–6) (Table 4, Fig. 6c). The NPS score was 85, indicating a high user satisfaction (Fig. 6d).

## Discussion

After organizing and constructing knowledge information, we found that the information items for robot-assisted surgery for PCa were redundant. RARPKB includes more than 2 million patients and 1589 cohorts from different regions, periods, and tumor characteristics. Prevailing research paradigms in PCa rely predominantly on rule-based or statistical methodologies that fail to adequately address the heterogeneity, complexity, and limitations of the disease associated with clinical expertise^40,41^. Facilitating timely access to standardized knowledge is critical for researchers and clinicians. In this era of flourishing AI, integrating AI and clinical medicine to intervene in PCa is an inevitable trend^42,43^, and intelligent medicine plays a pivotal role^44^. Thus, we present RARPKB, an Internet platform combining AI and human intelligence, to provide a readily accessible knowledge database on robot-assisted surgical interventions for PCa, catering to diverse potential user groups.

To our best knowledge, RARPKB is the pioneering knowledge-guided decision support platform dedicated to surgical interventions for PCa, marking its distinction as the first-ever knowledge database solely focused on robot-assisted surgery. By adhering to rigorous standardization, RARPKB diligently provides users with precise and comprehensive data on RARP for PCa.

ChatGPT-4, the apex of an AI chatbot, encompasses several clinical arenas^45^. The ChatGPT of AI is a preprocessing generative model. However, there are problems with the application of these models in medicine. First, owing to the current existence of more than 8000 highly complex diseases, universal AI models such as ChatGPT have sufficient breadth but insufficient depth and are not suitable for the precise surgical treatment of diseases, such as PCa^46,47^. The second point is that PCa is constantly evolving, and preprocessing models cannot be trained using future medical knowledge. They are also large in volume and have long update cycles and lags^48,49^. Thirdly, AI lacks precision, interpretability, and trustworthiness in the medical field^50^. Clinicians need precise knowledge and guidance, especially regarding invasive RARP operations^51^. Considering the complexity of the medical field and the substantial impact of clinical decision-making on an individual’s life, establishing an evidence-based and personalized knowledge platform-assisted decision-making system is crucial for achieving personalized treatment. We conducted a comparative analysis using simulated patient data, and the results definitively accentuate the specialized acumen of the RARPKB.

We employed 15 patient records as test cases to comprehensively compare the capabilities of ChatGPT and RARPKB in generating clinical scientific evidence for personalized surgical decision support. We assessed the evidence matching capability at basic, advanced, and professional categories (Table 3). The results indicated that both RARPKB and ChatGPT achieved a 100% accuracy rate in recommending the RARP surgical approach, demonstrating their success in providing patient-specific recommendations. RARPKB marginally outperformed ChatGPT in the authenticity and relevance of publication evidence, indicating the precision of evidence in RARPKB, while ChatGPT was prone to offering erroneous information. In other aspects such as personalized recommendations, patient matching, novelty of publication, and output standardization, RARPKB was superior to ChatGPT. This may be attributed to ChatGPT’s tendency to produce misinformation and knowledge hallucinations, as noted in the publication^52^. A recent study suggested that integrating clinical precision knowledge bases and knowledge graphs might mitigate this issue, a conclusion supported by our findings^53^. Despite the potential biases in testing and comparing RARPKB and ChatGPT-4 using data from 15 selected patients, we still conducted a comprehensive and rigorous evaluation to ensure the authenticity and validity of the research evidence. For specific issues pertaining to particular diseases, disease-specific knowledge bases demonstrate significant advantages and potential due to their precision and well-supported evidence, compared to general large-scale models. However, we advocate for future research with larger sample sizes to provide additional evidence to support this claim. While ChatGPT-4 functions as a versatile medical language model, RARPKB, a fusion of AI and human intelligence, distinguishes itself in the specialized realm of robotic-assisted surgery for PCa. In our next series of work, combining the two could potentially compensate for their respective shortcomings, thereby synergizing in both breadth and depth. At the same time, leveraging the effective evidence from RARPKB could address the general large model’s lack of interpretability and trustworthiness. Together, they are committed to advancing the practice of AI in PCa robotic surgery, aspiring to transform medical practice in this field^54^.

Some repositories cover various types of ailments. For instance, the Non-Alcoholic Fatty Liver Disease Knowledge Base is a specialized platform designed to assist in computational drug discovery for nonalcoholic fatty liver disease^55^. Although this platform provides comprehensive information, it focuses predominantly on drug mechanisms and fundamental research, and often overlooks clinical practice. Furthermore, it lacks a platform for user interaction, for example, a discussion forum. Similarly, the Pharmacogenomics Knowledge Base^56^ prioritizes research in pharmacogenomics. Although relevant to diseases, these studies remained focused on the genetic level and rarely considered clinical treatments. Repositories dedicated to surgical operations are lacking. Bridging the gap between genetics-level understanding and clinical practice is a lengthy process^57^. Hence, a knowledge base such as the RARPKB, which focuses on clinical practice, can directly support clinical work and decision-making. There is a substantial knowledge gap in this area, particularly for diseases such as PCa, which considerably affect males.

Gao *et al*.^58^ constructed a knowledge graph to delineate the association between cancer and obesity. This research archived two decades of data on cancer and obesity, including PCa. The authors’ construction standards and literature inclusion criteria were similar to ours. However, they built a visual relationship graph, which was essentially a large-scale semantic network of entities and relationships^59,60^. Nevertheless, their analysis was confined to bibliometric analysis rather than integrating knowledge information from original research into the database. The graph shows the annual publication numbers, regional and author analyses, keywords, organizations, citations, and other bibliometric aspects without being developed into a user-friendly web platform. Moreover, their research primarily focuses on the content of bibliometrics rather than clinical application practices. However, our work not only encompasses the field of bibliometrics but also places a strong emphasis on clinical application. We have established an online platform and developed clinical support tools to assist healthcare professionals, patients, and researchers in addressing practical issues.

Furthermore, RARPKB holds considerable value as a reference for personalized treatments. Within the context of intelligent medicine, precision medicine and personalized treatments are gradually becoming trends in future disease treatment^40,61^. Precise treatment plans and meticulous surgical procedures are crucial for anatomically intricate structures, such as the prostate. The comprehensive risk factor database for neurodegenerative diseases (NDDRF) is one such database focused on the personalized prevention of neurodegenerative diseases^62^. Comparable to our knowledge base, the NDDRF offers structured information and resources on neurodegenerative diseases, both of which have the potential to be employed for explainable AI modeling of diseases and the provision of personalized intervention. However, RARPKB specifically targets the surgical treatment of PCa and features advanced search and interaction functions.

RARPKB focuses on surgical interventions for PCa, providing immediate utility in clinical scenarios and valuable benchmarks for decision-making. Unlike existing databases that concentrate on genomics and require many steps for clinical translation, our knowledge base is distilled from actual clinical data and encompasses surgical methodologies. Consequently, it provides unambiguous guidelines for clinicians when considering surgical options. Validated through surveys of prospective users, RARPKB’s applied worth and future promise has become evident. More critically, as an easily accessible online resource, it equalizes healthcare by mitigating disparities in clinical experience^63^. Thus, integrated with human expertise, this AI-augmented database holds substantive merit for guiding clinical choices in PCa treatment.

Our research still has some limitations. First, the clinical issues of PCa are very complex, and our knowledge cannot cover all clinical situations. Nevertheless, we will continue to update and improve its information and functions in the future. Second, we only validated RARPKB through the responses from 15 inquiries, which has potential bias. Despite our best efforts to ensure that data and knowledge were solely collected from PubMed, future adjustments should include more sources of knowledge, such as Google Scholar and Web of Science. Additionally, we hope to survey RARPKB users, especially urologists, to further test and verify its clinical effectiveness, thereby optimizing the use of RARPKB and enhancing its clinical utility.

Knowledge bases are essential for modeling knowledge graphs and explainable AI models^64^. Therefore, RARPKB emphasized building a knowledge base in its current version. In the forthcoming version, we plan to build a knowledge graph and other explainable AI models based on this knowledge base. We also aim to construct an intelligent chatbot and clinical decision support system for robot-assisted PCa surgery^65^. In subsequent database updates, we intend to improve the data diversity and quality. These data, which are expected to be regularly updated, will enrich the database. On the basis of this knowledge, we plan to apply AI to an interactive intelligent chatbot to facilitate interactive question-and-answer sessions with users. In the future, we will integrate ontology, databases, knowledge graphs, and accurate clinical data to form a large-scale language model with credibility, logical clarity, and interpretability, thereby providing strong support for clinical decision-making.

## Conclusions

RARPKB constitutes the first knowledge base for robotic surgical treatment and provides insights into the clinical applications of radical prostatectomy and robot-assisted surgical systems in PCa treatment. As a medical knowledge resource, RARPKB has the potential to facilitate precise treatment and personalized surgical approaches in patients with PCa. Leveraging the knowledge database for knowledge graph construction and other interpretable AI models is necessary for future iterations.

## Ethical approval

Not applicable. The study does not involve patients.

## Consent

Not applicable.

## Sources of funding

This work was supported by “The Fundamental Research Funds for the Central Universities”. (No. 2023SCU12057), National Natural Science Foundation of China (Grant No. 32070671 and 32270690) and the regional innovation cooperation between Sichuan and Guangxi provinces (Project No. 2020YFQ0019).

## Author contribution

J.L. had full access to and verified all data used in this study and takes responsibility for the integrity of the data and the accuracy of the analysis. The authors confirm the following contribution to the final manuscript: J.L., E.W., T.Ta., L.Y., Q.W., and B.S.: study conception and design. J.L., J.Z., H.Z., R.W., Y.Q., Z.S., D.W., S.R., and Y.Q.: data collection. T.T., W.F., C.Z., and K.Z.: analysis and interpretation of results. J.L., T.T., E.W., J.Z., H.Z. and R.W.: draft manuscript preparation. All authors reviewed the results and approved the final version of the author disclosure form manuscript.

## Conflict of interest disclosure

Not applicable.

## Research registration unique identifying number (UIN)

Not applicable.

## Guarantor

Jiakun Li and Bairong Shen.

## Data availability statement

Data sharing is not applicable to this article.

## Provenance and peer review

Not commissioned, externally peer-reviewed.

## Supplementary Material

**Figure:**