# Supplementary material for: RARPKB: a knowledge-guide decision support platform for personalized robot-assisted surgery in prostate cancer
Source: Int J Surg. 2024 Mar 18;110(6):3412–24. doi: 10.1097/JS9.0000000000001290 (PMC11175739; doi:10.1097/JS9.0000000000001290)

**ChatGPT-4：**

TEST 1

Question:

I have some information about prostate cancer patients. Is this patient suitable for robot assisted radical prostatectomy. If you want to accept it, can you recommend some research literature that includes information about this patient in my research population? The information is: Age=65, BMI=30, PSA=5

What are the possible complications of this prostate cancer patient's robot assisted radical prostatectomy? Please also attach the research literature that matches the information of this patient.

ChatGPT-4:

Suitability for Robot Assisted Radical Prostatectomy (RARP):

Based on the information provided (Age=65, BMI=30, PSA=5):

1. Age (65 years): Age alone is not a contraindication for RARP. However, a thorough assessment of the patient's overall health, comorbidities, and fitness for surgery is essential. Many men in their 60s and even 70s can be suitable candidates if they are otherwise healthy.

2. BMI (30): A BMI of 30 places the patient in the obese category. Obesity can pose technical challenges during surgery, such as longer operative times and difficulties in achieving optimal visualization. However, with an experienced surgeon, RARP can still be safely performed in obese patients.

3. PSA (5): A PSA of 5 is moderately elevated. The PSA level helps to determine the likelihood of prostate cancer, its aggressiveness, and potential extent of spread. The decision for surgery would depend on further diagnostic procedures, like a biopsy, to confirm the presence and grade of cancer.

Research Literature:

For specific patient profiles like the one described, large-scale, randomized controlled trials might not be available. However, retrospective studies, case series, or reviews may provide insights. Here are some literature references you might consider:

1. Tewari A, Sooriakumaran P, Bloch DA, Seshadri-Kreaden U, Hebert AE, Wiklund P. Positive surgical margin and perioperative complication rates of primary surgical treatments for prostate cancer: a systematic review and meta-analysis comparing retropubic, laparoscopic, and robotic prostatectomy. Eur Urol. 2012;62(1):1-15.

2. Yaxley JW, Coughlin GD, Chambers SK, et al. Robot-assisted laparoscopic prostatectomy versus open radical retropubic prostatectomy: early outcomes from a randomised controlled phase 3 study. Lancet. 2016;388(10049):1057-1066.

3. Ploussard G, de la Taille A. The role of robot-assisted radical prostatectomy and pelvic lymph node dissection in the management of high-risk prostate cancer: a systematic review. Eur Urol. 2014;65(5):918-927.

Possible Complications of RARP:

Some potential complications of robot-assisted radical prostatectomy include:

1. Urinary incontinence: This is a common side effect, but most men regain bladder control over time.

2. Erectile dysfunction: The risk depends on factors like age, baseline function, and whether nerve-sparing techniques are used.

3. Bleeding and blood transfusion

4. Surgical site infection

5. Anesthesia complications

6. Deep vein thrombosis

7. Positive surgical margins: Indicating that not all of the cancer may have been removed.

8. Bladder neck contracture: Scarring at the bladder-urethra connection.

9. Ureteral injury

10. Lymphocele: Accumulation of lymphatic fluid, especially if lymph nodes are removed.

Research Literature on Complications:

To understand the complications in depth, consider reviewing:

1. Ficarra V, Novara G, Rosen RC, et al. Systematic review and meta-analysis of studies reporting urinary continence recovery after robot-assisted radical prostatectomy. Eur Urol. 2012;62(3):405-417.

2. Gandaglia G, Sammon JD, Chang SL, et al. Comparative effectiveness of robot-assisted and open radical prostatectomy in the postdissemination era. J Clin Oncol. 2014;32(14):1419-1426.

3. Menon M, Shrivastava A, Tewari A, et al. Laparoscopic and robot assisted radical prostatectomy: establishment of a structured program and preliminary analysis of outcomes. J Urol. 2002;168(3):945-949.

It's crucial to consult with an experienced urologist to make a well-informed decision regarding treatment. The literature provided can offer some context, but individual patient factors will heavily influence the decision and potential outcomes.

TEST 2

Question:

I have some information about prostate cancer patients. Is this patient suitable for robot assisted radical prostatectomy. If you want to accept it, can you recommend some research literature that includes information about this patient in my research population? The information is: Age=60, BMI=24, PSA=10

What are the possible complications of this prostate cancer patient's robot assisted radical prostatectomy? Please also attach the research literature that matches the information of this patient.

ChatGPT-4:

Suitability for Robot Assisted Radical Prostatectomy (RARP):

For the information provided (Age=60, BMI=24, PSA=10):

1. Age (60 years): Age by itself is not a contraindication for RARP. In fact, 60 years old is within the typical age range for men undergoing this procedure. The patient's overall health and other comorbidities should be evaluated.

2. BMI (24): A BMI of 24 is considered normal weight, which means the patient does not have obesity-related risks associated with the procedure. This is favorable for RARP.

3. PSA (10): A PSA of 10 is elevated and suggests a higher likelihood of prostate cancer. However, PSA alone does not confirm the aggressiveness or stage of the cancer. A biopsy is crucial to determine the presence, grade, and extent of cancer.

Research Literature:

The following studies and reviews may help provide insights into the suitability and outcomes of RARP for patients with similar characteristics:

1. Tewari A, Sooriakumaran P, Bloch DA, Seshadri-Kreaden U, Hebert AE, Wiklund P. Positive surgical margin and perioperative complication rates of primary surgical treatments for prostate cancer: a systematic review and meta-analysis comparing retropubic, laparoscopic, and robotic prostatectomy. Eur Urol. 2012;62(1):1-15.

2. Yaxley JW, Coughlin GD, Chambers SK, et al. Robot-assisted laparoscopic prostatectomy versus open radical retropubic prostatectomy: early outcomes from a randomised controlled phase 3 study. Lancet. 2016;388(10049):1057-1066.

3. Ficarra V, Novara G, Ahlering TE, et al. Systematic review and meta-analysis of studies reporting potency rates after robot-assisted radical prostatectomy. Eur Urol. 2012;62(3):418-430.

Possible Complications of RARP:

Potential complications include:

1. Urinary incontinence: A common side effect, but many men regain bladder control over time.

2. Erectile dysfunction: Depends on factors like age, baseline function, and nerve-sparing techniques.

3. Bleeding and blood transfusion

4. Surgical site infection

5. Anesthesia complications

6. Deep vein thrombosis

7. Positive surgical margins: Indicating potential residual cancer.

8. Bladder neck contracture

9. Ureteral injury

10. Lymphocele: Especially if lymph nodes are removed.

Research Literature on Complications:

To delve deeper into the complications and outcomes, consider the following literature:

1. Ficarra V, Novara G, Rosen RC, et al. Systematic review and meta-analysis of studies reporting urinary continence recovery after robot-assisted radical prostatectomy. Eur Urol. 2012;62(3):405-417.

2. Gandaglia G, Sammon JD, Chang SL, et al. Comparative effectiveness of robot-assisted and open radical prostatectomy in the postdissemination era. J Clin Oncol. 2014;32(14):1419-1426.

3. Menon M, Shrivastava A, Kaul S, et al. Vattikuti Institute prostatectomy: contemporary technique and analysis of results. Eur Urol. 2007;51(3):648-657; discussion 657-658.

As always, it's essential to consult with a specialized urologist for the best advice regarding treatment, especially given individual factors that can influence decision-making and outcomes.

TEST 3

Question:

I have some information about prostate cancer patients. Is this patient suitable for robot assisted radical prostatectomy. If you want to accept it, can you recommend some research literature that includes information about this patient in my research population? The information is: Age=50, BMI=22, PSA=4

What are the possible complications of this prostate cancer patient's robot assisted radical prostatectomy? Please also attach the research literature that matches the information of this patient.

ChatGPT-4:

Suitability for Robot Assisted Radical Prostatectomy (RARP):

Given the information provided (Age=50, BMI=22, PSA=4):

1. Age (50 years): This patient is relatively young, which might mean fewer comorbidities and better post-operative recovery. RARP can be a suitable option for younger men if prostate cancer is confirmed and other clinical parameters suggest a need for radical treatment.

2. BMI (22): A BMI of 22 falls within the normal weight range. This suggests the patient doesn't have obesity-related surgical risks, which is favorable for RARP.

3. PSA (4): A PSA of 4 is at the upper limit of what's typically considered the normal range. A PSA value in this range warrants further evaluation, especially with a prostate biopsy, to determine the presence and aggressiveness of any prostate cancer.

Research Literature:

The following studies and reviews might provide insights relevant to the patient's profile:

1. Tewari A, Sooriakumaran P, Bloch DA, Seshadri-Kreaden U, Hebert AE, Wiklund P. Positive surgical margin and perioperative complication rates of primary surgical treatments for prostate cancer: a systematic review and meta-analysis comparing retropubic, laparoscopic, and robotic prostatectomy. Eur Urol. 2012;62(1):1-15.

2. Yaxley JW, Coughlin GD, Chambers SK, et al. Robot-assisted laparoscopic prostatectomy versus open radical retropubic prostatectomy: early outcomes from a randomised controlled phase 3 study. Lancet. 2016;388(10049):1057-1066.

3. Ficarra V, Novara G, Rosen RC, et al. Systematic review and meta-analysis of studies reporting potency rates after robot-assisted radical prostatectomy. Eur Urol. 2012;62(3):418-430.

Possible Complications of RARP:

Potential complications include:

1. Urinary incontinence: A common side effect, though younger men tend to have better rates of recovery.

2. Erectile dysfunction: The risk is influenced by age, baseline function, surgical technique, and other factors.

3. Bleeding and blood transfusion

4. Surgical site infection

5. Anesthesia complications

6. Deep vein thrombosis

7. Positive surgical margins: Indicating potential residual cancer.

8. Bladder neck contracture

9. Ureteral injury

10. Lymphocele: Especially if lymph nodes are removed.

Research Literature on Complications:

For an understanding of complications and outcomes, you might consider:

1. Ficarra V, Novara G, Rosen RC, et al. Systematic review and meta-analysis of studies reporting urinary continence recovery after robot-assisted radical prostatectomy. Eur Urol. 2012;62(3):405-417.

2. Gandaglia G, Sammon JD, Chang SL, et al. Comparative effectiveness of robot-assisted and open radical prostatectomy in the postdissemination era. J Clin Oncol. 2014;32(14):1419-1426.

3. Menon M, Shrivastava A, Kaul S, et al. Vattikuti Institute prostatectomy: contemporary technique and analysis of results. Eur Urol. 2007;51(3):648-657; discussion 657-658.

As always, individual patient considerations play a key role. It's crucial to collaborate with an experienced urologist to assess the best course of action for any patient.

TEST 4

Question:

I have some information about prostate cancer patients. Is this patient suitable for robot assisted radical prostatectomy. If you want to accept it, can you recommend some research literature that includes information about this patient in my research population? The information is: Age=40, BMI=26, PSA=5.5. What are the possible complications of this prostate cancer patient's robot assisted radical prostatectomy? Please also attach the research literature that matches the information of this patient.

ChatGPT-4:

I'm not a healthcare professional, but I can provide some general information based on the parameters you've given. The decision of whether a patient is suitable for robot-assisted radical prostatectomy ultimately needs to be made by qualified medical professionals who are familiar with the patient's full medical history and condition.

Criteria for Robot-Assisted Radical Prostatectomy

1. Age: The patient is relatively young at 40, which is generally favorable for surgery, but age is only one factor to consider.

2. BMI: A BMI of 26 is considered overweight but not obese, so it may not be a significant barrier for surgery.

3. PSA: A PSA level of 5.5 ng/mL is elevated and may be indicative of prostate cancer, although other conditions can also elevate PSA. Biopsy and imaging studies are crucial for diagnosis and staging.

Research Literature

For your research, you might find the following studies and review articles helpful in evaluating this patient's suitability for robot-assisted radical prostatectomy:

1. "Robot-assisted radical prostatectomy: a review of outcomes" published in BJU International.

2. "Factors influencing the choice of surgical approach for patients with localized prostate cancer" published in the Journal of Urology.

3. "Age, Body Mass Index, and Prostate-Specific Antigen as Determinants for Robot-Assisted Radical Prostatectomy" published in the Journal of Endourology.

Possible Complications

1. Bleeding: Even though the risk is lower in robotic surgery compared to open surgery, bleeding is a possibility.

2. Infection: Infections can occur post-surgery.

3. Urinary Incontinence: This is a common issue after any form of prostatectomy.

4. Erectile Dysfunction: Surgery can affect sexual function, although nerve-sparing techniques aim to mitigate this.

5. Positive Surgical Margins: There's a risk that not all cancer will be removed.

6. Anesthesia Risks: As with any surgery requiring general anesthesia, there are risks involved.

Consult with a healthcare provider for the most accurate and personalized advice.

TEST 5

Question:

I have some information about prostate cancer patients. Is this patient suitable for robot assisted radical prostatectomy. If you want to accept it, can you recommend some research literature that includes information about this patient in my research population? The information is: Age=80, BMI=32, PSA=8

What are the possible complications of this prostate cancer patient's robot assisted radical prostatectomy? Please also attach the research literature that matches the information of this patient.

ChatGPT-4:

Suitability for Robot Assisted Radical Prostatectomy (RARP):

For the provided information (Age=80, BMI=32, PSA=8):

1. Age (80 years): At 80, the patient is older than most men who typically undergo RARP. Age is a significant factor to consider due to potential comorbidities and slower recovery. Many urologists might lean towards conservative management, like active surveillance or radiation, unless the cancer is aggressive or symptomatic.

2. BMI (32): A BMI of 32 indicates obesity. Obesity can increase surgical complications and can affect recovery.

3. PSA (8): A PSA of 8 is elevated. However, considering the patient's age, the PSA density (PSA divided by prostate volume) and the results of a biopsy would provide a more comprehensive understanding of the cancer's aggressiveness.

Research Literature:

For older patients undergoing RARP:

1. Trinh QD, Sammon J, Sun M, et al. Perioperative outcomes of robot-assisted radical prostatectomy compared with open radical prostatectomy: results from the nationwide inpatient sample. Eur Urol. 2012;61(4):679-685.

2. Dalela D, Jeong W, Prasad MA, et al. A Pragmatic Randomized Controlled Trial Examining the Impact of the Retzius-sparing Approach on Early Urinary Continence Recovery after Robot-assisted Radical Prostatectomy. Eur Urol. 2017;72(5):677-685.

3. Resnick MJ, Koyama T, Fan KH, et al. Long-term functional outcomes after treatment for localized prostate cancer. N Engl J Med. 2013;368(5):436-445.

Possible Complications of RARP:

For an older patient with a higher BMI:

1. Urinary incontinence: Risk may be higher in older men.

2. Erectile dysfunction: Age, surgical technique, and other factors play roles in post-operative erectile function.

3. Bleeding and blood transfusion

4. Surgical site infection: Obesity can increase the risk.

5. Anesthesia complications: Especially relevant in older patients with potential cardiac or respiratory issues.

6. Deep vein thrombosis: Obesity and age can increase the risk.

7. Positive surgical margins

8. Bladder neck contracture

9. Ureteral injury

10. Lymphocele

11. Delayed recovery: Due to age and obesity.

Research Literature on Complications:

For complications, especially in older men or those with higher BMIs:

1. Gandaglia G, Sammon JD, Chang SL, et al. Comparative effectiveness of robot-assisted and open radical prostatectomy in the postdissemination era. J Clin Oncol. 2014;32(14):1419-1426.

2. Tewari A, Sooriakumaran P, Bloch DA, Seshadri-Kreaden U, Hebert AE, Wiklund P. Positive surgical margin and perioperative complication rates of primary surgical treatments for prostate cancer: a systematic review and meta-analysis comparing retropubic, laparoscopic, and robotic prostatectomy. Eur Urol. 2012;62(1):1-15.

3. Ploussard G, de la Taille A. The role of robot-assisted radical prostatectomy and pelvic lymph node dissection in the management of high-risk prostate cancer: a systematic review. Eur Urol. 2014;66(5):918-927.

In conclusion, the decision to undergo RARP should be individualized, taking into account the patient's overall health, life expectancy, cancer grade and stage, and the potential risks and benefits of the procedure. It's crucial to discuss the situation with an experienced urologist or uro-oncologist.

TEST 6

Question:

I have some information about prostate cancer patients. Is this patient suitable for robot assisted radical prostatectomy. If you want to accept it, can you recommend some research literature that includes information about this patient in my research population? The information is: Age=62, BMI=25, PSA=10 T stage=2b, bGS=3+4.

What are the possible complications of this prostate cancer patient's robot assisted radical prostatectomy? Please also attach the research literature that matches the information of this patient.

ChatGPT-4:

Suitability for Robot Assisted Radical Prostatectomy (RARP):

For the provided information (Age=62, BMI=25, PSA=10, T stage=2b, Gleason Score 3+4):

1. Age (62 years): The patient's age is within the range where RARP is commonly considered, but age is only one factor in the decision-making process.

2. BMI (25): A BMI of 25 is considered to be on the upper end of normal weight, so obesity isn't a concern for surgical complications in this case.

3. PSA (10): A PSA of 10 is elevated, suggesting prostate cancer.

4. T stage (2b): This indicates the cancer is present in more than half of one lobe of the prostate but hasn't spread to other nearby tissues.

5. Gleason Score (3+4): This gives a total score of 7, indicating moderately aggressive cancer.

Based on the provided data, the patient is a potential candidate for RARP. However, individual clinical and patient factors, as well as personal preferences, must be considered.

Research Literature:

1. Tewari A, Sooriakumaran P, Bloch DA, Seshadri-Kreaden U, Hebert AE, Wiklund P. Positive surgical margin and perioperative complication rates of primary surgical treatments for prostate cancer: a systematic review and meta-analysis comparing retropubic, laparoscopic, and robotic prostatectomy. Eur Urol. 2012;62(1):1-15.

2. Yaxley JW, Coughlin GD, Chambers SK, et al. Robot-assisted laparoscopic prostatectomy versus open radical retropubic prostatectomy: early outcomes from a randomised controlled phase 3 study. Lancet. 2016;388(10049):1057-1066.

3. Ficarra V, Novara G, Rosen RC, et al. Systematic review and meta-analysis of studies reporting urinary continence recovery after robot-assisted radical prostatectomy. Eur Urol. 2012;62(3):405-417.

Possible Complications of RARP:

1. Urinary incontinence: Potential risk due to disruption of the urinary sphincter and surrounding structures.

2. Erectile dysfunction: This can be due to injury or disruption of the nerve bundles responsible for erections.

3. Bleeding and blood transfusion

4. Surgical site infection

5. Anesthesia complications

6. Deep vein thrombosis

7. Positive surgical margins: Indicates that cancer cells might be left behind.

8. Bladder neck contracture: Scarring that can block the flow of urine.

9. Ureteral injury

10. Lymphocele: Collection of lymph fluid in the pelvis after removal of lymph nodes.

Research Literature on Complications:

1. Gandaglia G, Sammon JD, Chang SL, et al. Comparative effectiveness of robot-assisted and open radical prostatectomy in the postdissemination era. J Clin Oncol. 2014;32(14):1419-1426.

2. Menon M, Shrivastava A, Kaul S, et al. Vattikuti Institute prostatectomy: contemporary technique and analysis of results. Eur Urol. 2007;51(3):648-657; discussion 657-658.

3. Loeb S, Carter HB, Berndt SI, Ricker W, Schaeffer EM. Complications after prostate biopsy: data from SEER-Medicare. J Urol. 2011;186(5):1830-1834.

While the patient's data suggests a potential for RARP, it's essential to weigh the risks and benefits of the procedure. The patient should discuss this in-depth with his urologist or uro-oncologist.

TEST 7

Question:

I have some information about prostate cancer patients. Is this patient suitable for robot assisted radical prostatectomy. If you want to accept it, can you recommend some research literature that includes information about this patient in my research population? The information is: Age=67, BMI=26.2 PSA=15.4, T stage=T2b, bGS=4+3, ASA=3, CCI=2.

What are the possible complications of this prostate cancer patient's robot assisted radical prostatectomy? Please also attach the research literature that matches the information of this patient.

ChatGPT-4:

Suitability for Robot Assisted Radical Prostatectomy (RARP):

Given the provided information (Age=67, BMI=26.2, PSA=15.4, T stage=T2b, Gleason Score 4+3, ASA=3, CCI=2):

1. Age (67 years): The age is within a typical range for patients who undergo RARP, but individual health considerations are paramount.

2. BMI (26.2): Slightly overweight but not necessarily a contraindication for RARP.

3. PSA (15.4): A significantly elevated PSA, which is consistent with prostate cancer.

4. T stage (T2b): This means the cancer is present in more than half of one lobe of the prostate but hasn't spread to other nearby tissues.

5. Gleason Score (4+3): This gives a total score of 7, which indicates a more aggressive form of cancer.

6. ASA 3: This American Society of Anesthesiologists score indicates the patient has severe systemic disease. This is an important consideration for surgery, as it could increase operative risk.

7. CCI (Charlson Comorbidity Index) of 2: Indicates the patient has some other comorbid conditions that may increase the risk of mortality.

The patient's data does suggest that RARP can be considered; however, the ASA and CCI scores indicate existing health concerns that need to be thoroughly evaluated and considered in the surgical decision-making process.

Research Literature:

1. Ficarra V, Novara G, Artibani W, et al. Retropubic, laparoscopic, and robot-assisted radical prostatectomy: a systematic review and cumulative analysis of comparative studies. Eur Urol. 2009;55(5):1037-1063.

2. Trinh QD, Sammon J, Sun M, et al. Perioperative outcomes of robot-assisted radical prostatectomy compared with open radical prostatectomy: results from the nationwide inpatient sample. Eur Urol. 2012;61(4):679-685.

3. Coelho RF, Palmer KJ, Rocco B, Moniz RR, Chauhan S, Orvieto MA, Coughlin G, Patel VR. Early complication rates in a single-surgeon series of 2500 robotic-assisted radical prostatectomies: report applying a standardized grading system. Eur Urol. 2010;57(6):945-952.

Possible Complications of RARP:

1. Urinary incontinence

2. Erectile dysfunction

3. Bleeding and blood transfusion

4. Surgical site infection

5. Anesthesia complications: Especially relevant given the ASA score of 3.

6. Deep vein thrombosis

7. Positive surgical margins

8. Bladder neck contracture

9. Ureteral injury

10. Lymphocele

11. Complications related to comorbid conditions: Given the CCI score.

Research Literature on Complications:

1. Gandaglia G, Sammon JD, Chang SL, et al. Comparative effectiveness of robot-assisted and open radical prostatectomy in the postdissemination era. J Clin Oncol. 2014;32(14):1419-1426.

2. Ficarra V, Novara G, Ahlering TE, et al. Systematic review and meta-analysis of studies reporting potency rates after robot-assisted radical prostatectomy. Eur Urol. 2012;62(3):418-430.

3. Loeb S, Vellekoop A, Ahmed HU, et al. Systematic review of complications of prostate biopsy. Eur Urol. 2013;64(6):876-892.

Given the patient's overall health status and the aggressiveness of the cancer, a comprehensive discussion with the urologist or uro-oncologist is necessary to assess the risks and benefits of the procedure.

TEST 8

Question:

I have some information about prostate cancer patients. Is this patient suitable for robot assisted radical prostatectomy. If you want to accept it, can you recommend some research literature that includes information about this patient in my research population? The information is: Age=58, BMI=26.8, PSA=5.6, T stage=T1 , bGS, 3+3, race, Asian.

What are the possible complications of this prostate cancer patient's robot assisted radical prostatectomy? Please also attach the research literature that matches the information of this patient.

ChatGPT-4:

Suitability for Robot Assisted Radical Prostatectomy (RARP):

Given the provided information (Age=58, BMI=26.8, PSA=5.6, T stage=T1, Gleason Score 3+3, Race=Asian):

1. Age (58 years): This age is suitable for RARP.

2. BMI (26.8): This BMI places the patient in the overweight category but shouldn't be a contraindication for RARP.

3. PSA (5.6): Elevated, but within a range that would be consistent with early prostate cancer or other benign conditions.

4. T stage (T1): This stage indicates cancer that cannot be felt during an examination and was identified accidentally, often during a treatment for another condition or through a PSA test.

5. Gleason Score (3+3): This gives a total score of 6, indicating the least aggressive form of prostate cancer.

6. Race (Asian): Some studies suggest that Asian men may have different outcomes or disease characteristics, but these results can be varied. This demographic factor should be considered in combination with clinical factors.

Considering the patient's information, RARP could be a suitable treatment option, especially if the patient and physician decide on definitive treatment over active surveillance, given the Gleason score of 6.

Research Literature:

1. Jeong W, Park SY, Lorenzo EI, et al. Robotic versus open radical prostatectomy in clinically localized prostate cancer: A comparison of oncological outcomes. Urol Oncol. 2016;34(3):121.e1-7.

2. Kim SP, Shah ND, Weight CJ, et al. Contemporary trends in nephron sparing surgery (NSS) versus radical nephrectomy for renal cell carcinoma. J Urol. 2011;186(5):1779-1785.

3. Wong MC, Goggins WB, Yip BH, et al. Incidence and mortality of prostate cancer: global, regional, and national estimates for 2012.” Asian Pac J Cancer Prev. 2016;17(4):2119-2126. – This provides insight into the prostate cancer trends among Asian populations.

Possible Complications of RARP:

1. Urinary incontinence: This can be due to disruption or injury to the urinary sphincter.

2. Erectile dysfunction: This can occur due to injury or disruption of the nerve bundles responsible for erections.

3. Bleeding and need for blood transfusion.

4. Surgical site infection.

5. Anesthesia-related complications.

6. Deep vein thrombosis or blood clots.

7. Positive surgical margins: Indicates cancer cells might be left behind.

8. Bladder neck contracture: Scarring that can block the flow of urine.

9. Ureteral injury.

10. Lymphocele: A collection of lymph fluid in the pelvis after removal of lymph nodes.

Research Literature on Complications:

1. Ficarra V, Novara G, Ahlering TE, et al. Systematic review and meta-analysis of studies reporting potency rates after robot-assisted radical prostatectomy. Eur Urol. 2012;62(3):418-430.

2. Yaxley JW, Coughlin GD, Chambers SK, et al. Robot-assisted laparoscopic prostatectomy versus open radical retropubic prostatectomy: early outcomes from a randomised controlled phase 3 study. Lancet. 2016;388(10049):1057-1066.

3. Loeb S, Carter HB, Berndt SI, Ricker W, Schaeffer EM. Complications after prostate biopsy: data from SEER-Medicare. J Urol. 2011;186(5):1830-1834.

The patient should discuss in-depth with his urologist or uro-oncologist to make an informed decision regarding treatment.

TEST 9

Question:

I have some information about prostate cancer patients. Is this patient suitable for robot assisted radical prostatectomy. If you want to accept it, can you recommend some research literature that includes information about this patient in my research population? The information is: Age=64, BMI=25.5, PSA=10.2, T stage=T2b, WHO/ISUP=5, CCI=3, risk=high.

What are the possible complications of this prostate cancer patient's robot assisted radical prostatectomy? Please also attach the research literature that matches the information of this patient.

ChatGPT-4:

Suitability for Robot Assisted Radical Prostatectomy (RARP):

Given the provided information (Age=64, BMI=25.5, PSA=10.2, T stage=T2b, WHO/ISUP=5, CCI=3, Risk=High):

1. Age (64 years): The age is within a typical range for patients who undergo RARP.

2. BMI (25.5): This is within the "Overweight" range but not a significant contraindication for RARP.

3. PSA (10.2): Elevated and consistent with prostate cancer.

4. T stage (T2b): Indicates cancer involvement in more than half of one side of the prostate.

5. WHO/ISUP grade (5): This is a very high grade indicating aggressive cancer.

6. CCI (Charlson Comorbidity Index) of 3: Indicates the patient has comorbid conditions that may increase the risk of mortality and post-operative complications.

7. Risk (High): Reflects that the patient has high-risk prostate cancer, often indicating aggressive disease that may benefit from definitive treatment.

Considering the aggressive nature of the cancer (based on the WHO/ISUP grade and the risk classification), RARP may be an appropriate option if the patient is deemed surgically fit given the CCI score.

Research Literature:

1. Ficarra V, Novara G, Artibani W, et al. Retropubic, laparoscopic, and robot-assisted radical prostatectomy: a systematic review and cumulative analysis of comparative studies. Eur Urol. 2009;55(5):1037-1063.

2. Menon M, Shrivastava A, Kaul S, et al. Vattikuti Institute prostatectomy: contemporary technique and analysis of results. Eur Urol. 2007;51(3):648-657.

3. Sundi D, Ross AE, Humphreys EB, et al. African American men with very low-risk prostate cancer exhibit adverse oncologic outcomes after radical prostatectomy: should active surveillance still be an option for them? J Clin Oncol. 2013;31(24):2991-2997.

Possible Complications of RARP:

1. Urinary incontinence

2. Erectile dysfunction

3. Bleeding and possible need for blood transfusion

4. Surgical site infection

5. Anesthesia complications

6. Deep vein thrombosis

7. Positive surgical margins

8. Bladder neck contracture or urethral stricture

9. Ureteral injury

10. Lymphocele formation after pelvic lymph node dissection

11. Potential complications from comorbidities: Given the CCI score.

Research Literature on Complications:

1. Gandaglia G, Sammon JD, Chang SL, et al. Comparative effectiveness of robot-assisted and open radical prostatectomy in the postdissemination era. J Clin Oncol. 2014;32(14):1419-1426.

2. Ficarra V, Novara G, Rosen RC, et al. Systematic review and meta-analysis of studies reporting urinary continence recovery after robot-assisted radical prostatectomy. Eur Urol. 2012;62(3):405-417.

3. Loeb S, Vellekoop A, Ahmed HU, et al. Systematic review of complications of prostate biopsy. Eur Urol. 2013;64(6):876-892.

Given the patient's high-risk cancer and other health factors, a comprehensive discussion with the urologist or uro-oncologist is necessary to evaluate the potential benefits and risks of the procedure.

TEST 10

Question:

I have some information about prostate cancer patients. Is this patient suitable for robot assisted radical prostatectomy. If you want to accept it, can you recommend some research literature that includes information about this patient in my research population? The information is: Age=71, BMI=24.1, PSA=20, T stage=T3, WHO/ISUP=4.

What are the possible complications of this prostate cancer patient's robot assisted radical prostatectomy? Please also attach the research literature that matches the information of this patient.

ChatGPT-4:

Suitability for Robot Assisted Radical Prostatectomy (RARP):

Given the provided information (Age=71, BMI=24.1, PSA=20, T stage=T3, WHO/ISUP=4):

1. Age (71 years): While age alone is not an absolute contraindication, older age may come with increased risk of complications and slower recovery.

2. BMI (24.1): This is a normal BMI and should not pose a significant concern.

3. PSA (20): A markedly elevated PSA, indicative of more aggressive or advanced disease.

4. T stage (T3): Indicates extraprostatic extension. The cancer has spread outside the prostate and may have invaded the seminal vesicles.

5. WHO/ISUP grade (4): High grade, indicating aggressive disease.

Given the aggressive nature of the cancer (based on the T stage, PSA, and WHO/ISUP grade), other treatments such as radiation therapy and hormone therapy might also be considered alongside or instead of surgery. If RARP is considered, it should be approached with an understanding of the potential benefits and limitations in the context of T3 disease.

Research Literature:

1. Tewari A, Sooriakumaran P, Bloch DA, et al. Positive Surgical Margin and Perioperative Complication Rates of Primary Surgical Treatments for Prostate Cancer: A Systematic Review and Meta-analysis Comparing Retropubic, Laparoscopic, and Robotic Prostatectomy. Eur Urol. 2012;62(1):1-15.

2. Yuh BE, Nazmy M, Ruel NH, et al. Standardized comparison of robot-assisted limited and extended pelvic lymphadenectomy for prostate cancer. BJU Int. 2013;112(1):81-88.

3. Cooperberg MR, Carroll PR. Trends in Management for Patients With Localized Prostate Cancer, 1990-2013. JAMA. 2015;314(1):80-82.

Possible Complications of RARP:

1. Urinary incontinence

2. Erectile dysfunction

3. Bleeding and potential need for blood transfusion

4. Surgical site infection

5. Anesthesia complications

6. Deep vein thrombosis

7. Positive surgical margins

8. Bladder neck contracture or urethral stricture

9. Ureteral injury

10. Lymphocele formation after pelvic lymph node dissection

Given the T3 stage, there's an increased risk of positive surgical margins, which may lead to the need for additional treatments postoperatively.

Research Literature on Complications:

1. Gandaglia G, Sammon JD, Chang SL, et al. Comparative Effectiveness of Robot-assisted and Open Radical Prostatectomy in the Postdissemination Era. J Clin Oncol. 2014;32(14):1419-1426.

2. Ficarra V, Novara G, Rosen RC, et al. Systematic review and meta-analysis of studies reporting urinary continence recovery after robot-assisted radical prostatectomy. Eur Urol. 2012;62(3):405-417.

3. Loeb S, Vellekoop A, Ahmed HU, et al. Systematic Review of Complications of Prostate Biopsy. Eur Urol. 2013;64(6):876-892.

Given the patient's T3 cancer and age, a detailed consultation with the urologist or uro-oncologist is vital to discuss the potential benefits and risks of the procedure, as well as other potential treatment modalities.

TEST 11

Question:

I am a urologist and have some information about a prostate cancer patient who underwent robot assisted radical prostatectomy. Can you recommend some research literature that includes information about this patient in my research population? The information is: Age=66, BMI=26.3, PSA=6.5, T2b, ISUP3, and the patient underwent an expanded Pelvic Lymph Node Dissection during the surgery, but not a lymph nodes removed, and underwent a Nerve sparing procedure.

What are the possible complications of this prostate cancer patient's robot assisted radical prostatectomy? Please also attach the research literature that matches the information of this patient.

ChatGPT-4:

Certainly. For a prostate cancer patient with the given profile (Age=66, BMI=26.3, PSA=6.5, T2b, ISUP3, expanded Pelvic Lymph Node Dissection without lymph nodes removed, and Nerve sparing procedure), here's a relevant selection of research literature:

Research Literature for the Given Patient Profile:

1. Ficarra V, Novara G, Ahlering TE, et al. Systematic review and meta-analysis of studies reporting potency rates after robot-assisted radical prostatectomy. Eur Urol. 2012;62(3):418-430. (This discusses potency outcomes, particularly relevant because of the nerve-sparing procedure.)

2. Tewari A, Sooriakumaran P, Bloch DA, et al. Positive Surgical Margin and Perioperative Complication Rates of Primary Surgical Treatments for Prostate Cancer: A Systematic Review and Meta-analysis Comparing Retropubic, Laparoscopic, and Robotic Prostatectomy. Eur Urol. 2012;62(1):1-15.

3. Yuh BE, Nazmy M, Ruel NH, et al. Standardized comparison of robot-assisted limited and extended pelvic lymphadenectomy for prostate cancer. BJU Int. 2013;112(1):81-88. (This will be particularly relevant given the expanded Pelvic Lymph Node Dissection mentioned.)

4. Cooperberg MR, Carroll PR. Trends in Management for Patients With Localized Prostate Cancer, 1990-2013. JAMA. 2015;314(1):80-82.

Possible Complications of RARP for the Patient:

1. Urinary incontinence: Given the nerve-sparing procedure, there may be a reduced risk, but it remains a potential post-operative complication.

2. Erectile dysfunction: The nerve-sparing procedure aims to preserve potency, but ED can still occur post-operatively.

3. Bleeding and potential need for blood transfusion

4. Surgical site infection

5. Anesthesia complications

6. Deep vein thrombosis

7. Positive surgical margins: Especially relevant for T2b disease.

8. Bladder neck contracture or urethral stricture

9. Ureteral injury

10. Lymphocele formation after pelvic lymph node dissection: Despite not having any nodes removed, the dissection can still lead to lymphocele.

TEST 12

Question:

I am a urologist and have some information about a prostate cancer patient who underwent robot assisted radical prostatectomy. Can you recommend some research literature that includes information about this patient in my research population? The information is: Age=65, BMI=25.5, PSA=7.2, T2b, ISUP2, and the patient not underwent an expanded Pelvic Lymph Node Dissection during the surgery, but not a lymph nodes removed, and not underwent a Nerve sparing procedure.

What are the possible complications of this prostate cancer patient's robot assisted radical prostatectomy? Please also attach the research literature that matches the information of this patient.

ChatGPT-4:

I understand that you're a healthcare professional looking for research literature to better understand this specific prostate cancer case. Here are some factors to consider, potential complications, and suggested literature:

Criteria for Robot-Assisted Radical Prostatectomy

1. Age: At 65 years, the patient is within the common age range for prostate cancer surgery.

2. BMI: A BMI of 25.5 falls within the 'overweight' range, but not obese, potentially reducing surgical risk.

3. PSA: A PSA of 7.2 ng/mL indicates a need for further diagnostic evaluation and treatment.

4. T2b: Indicates that cancer is in more than one-half of one side of the prostate but not both sides.

5. ISUP Grade 2: A moderate grade, suggesting that the cancer is not extremely aggressive but still requires attention.

6. No ePLND and No Nerve Sparing: This could imply higher risk factors or other comorbidities that prevented these procedures.

Recommended Research Literature

1. "Robot-assisted radical prostatectomy in men aged >65 years" published in BJU International - A study focusing on older adults undergoing robot-assisted radical prostatectomy.

2. "Effect of Body Mass Index on Robotic-assisted Radical Prostatectomy" published in the Journal of Endourology - This article specifically discusses BMI's impact on surgical outcomes.

3. "Prognostic value of the new Grade Groups in Prostate Cancer: a multi-institutional European validation study" published in the European Urology Journal - Focuses on ISUP grades and their prognostic value.

4. "Surgical margin status after robot assisted radical prostatectomy: a multi-institutional study" published in Journal of Urology - Relevant for the understanding of cancer clearance in absence of nerve-sparing and ePLND.

Possible Complications

1. Bleeding: Even with robotic assistance, some risk of bleeding persists.

2. Infection: Post-operative infection is a risk, especially in older adults.

3. Urinary Incontinence: This is more likely due to the absence of nerve-sparing procedures.

4. Erectile Dysfunction: Also more likely due to the absence of nerve-sparing procedures.

5. Positive Surgical Margins: There's always a risk that some cancer may remain.

6. Lymphedema: Since no ePLND was done, this risk is likely lower, but it can still occur.

7. Anesthesia Risks: Older adults may have increased risk factors related to anesthesia.

I recommend consulting these and other papers for the most comprehensive and personalized evaluation.

TEST 13

Question:

I am a urologist and have some information about a prostate cancer patient who underwent robot assisted radical prostatectomy. Can you recommend some research literature that includes information about this patient in my research population? The information is: Age=67, BMI=26.4, PSA=18.4, T2c, ISUP3, and the patient underwent an expanded Pelvic Lymph Node Dissection during the surgery, but not a lymph nodes removed, and underwent a Nerve sparing procedure.

What are the possible complications of this prostate cancer patient's robot assisted radical prostatectomy? Please also attach the research literature that matches the information of this patient.

ChatGPT-4:

Certainly, based on the information provided, I can guide you towards research literature that may be pertinent to this specific patient profile.

Criteria for Robot-Assisted Radical Prostatectomy

1. Age: The patient, at 67 years, is within the common age range for prostate cancer surgery.

2. BMI: A BMI of 26.4 categorizes the patient as overweight, which could have certain implications for the procedure.

3. PSA: A PSA of 18.4 ng/mL is considerably elevated, indicating a higher risk category.

4. T2c: This stage indicates that cancer is present in both sides of the prostate.

5. ISUP Grade 3: This grade suggests an intermediate risk, implying that the cancer cells are moderately differentiated.

6. ePLND Done: Expanded Pelvic Lymph Node Dissection was done, but no lymph nodes were removed, possibly suggesting no macroscopic nodal involvement.

7. Nerve Sparing Done: This procedure can help in preserving urinary continence and sexual function postoperatively.

Recommended Research Literature

1. Tewari, A. et al. "Long-term survival probability in men with clinically localized prostate cancer: a case-control, propensity modeling study stratified by race, age, treatment, and comorbidities." The Journal of urology.

2. Loeb, S., et al. "Systematic review of complications of prostate biopsy." European Urology.

3. Carlsson, S., et al. "Robot-assisted radical prostatectomy for clinically localized prostate cancer: A systematic review and meta-analysis." European Urology.

4. Abdollah, F., et al. "Pelvic Lymph Node Dissection for Patients with Elevated Risk of Lymph Node Invasion during Radical Prostatectomy: Comparison of Open, Laparoscopic and Robot-assisted Procedures." Journal of Endourology.

5. Menon, M., et al. "Prospective assessment of short-term impact and recovery of health-related quality of life in men undergoing robotic radical prostatectomy." European Urology.

Possible Complications

1. Bleeding: Even with robotic assistance, some risk of bleeding remains.

2. Infection: Post-operative infection is a concern.

3. Urinary Incontinence: Although nerve-sparing techniques reduce this risk, there's still a possibility, especially in the immediate post-operative period.

4. Erectile Dysfunction: The risk is diminished by nerve-sparing techniques but cannot be entirely ruled out.

5. Positive Surgical Margins: This could suggest residual cancer and might require further treatment.

6. Lymphocele Formation: Given the ePLND, fluid collection in the pelvis can occur.

7. Anesthesia Risks: Standard risks associated with the use of anesthesia during surgery.

Please refer to the mentioned literature for more detailed information regarding potential complications and outcomes related to the provided patient profile. Always ensure that you are referencing the most current and up-to-date research in your clinical decisions.

TEST 14

Question:

I am a urologist and have some information about a prostate cancer patient who underwent robot assisted radical prostatectomy. Can you recommend some research literature that includes information about this patient in my research population? The information is: Age=59, BMI=29.5, PSA=8.6, T2b, bGS3+5. And the patient did not undergo an expanded Pelvic Limph Node Dissection, a Limph nodes removed, and a Nerve sparing procedure during the surgery.

What are the possible complications of this prostate cancer patient's robot assisted radical prostatectomy? Please also attach the research literature that matches the information of this patient.

ChatGPT-4:

For a prostate cancer patient with the following characteristics (Age=59, BMI=29.5, PSA=8.6, T2b, bGS 3+5, and no expanded Pelvic Lymph Node Dissection, no lymph nodes removed, and nerve-sparing procedure during the surgery), I can provide a selection of relevant research literature and discuss possible complications after robot-assisted radical prostatectomy (RARP).

Research Literature for the Given Patient Profile:

1. Tewari A, Sooriakumaran P, Bloch DA, Seshadri-Kreaden U, Hebert AE, Wiklund P. Positive Surgical Margin and Perioperative Complication Rates of Primary Surgical Treatments for Prostate Cancer: A Systematic Review and Meta-analysis Comparing Retropubic, Laparoscopic, and Robotic Prostatectomy. Eur Urol. 2012;62(1):1-15.

- Relevant for understanding the implications of positive surgical margins and the rate of complications after RARP.

2. Ficarra V, Novara G, Ahlering TE, et al. Systematic review and meta-analysis of studies reporting urinary continence recovery after robot-assisted radical prostatectomy. Eur Urol. 2012;62(3):405-417.

- Given the nerve-sparing procedure, understanding post-surgery urinary continence recovery is crucial.

3. Coughlin GD, Yaxley JW, Chambers SK, et al. Robot-assisted laparoscopic prostatectomy versus open radical retropubic prostatectomy: 24-month outcomes from a randomised controlled study. Lancet Oncol. 2018;19(8):1051-1060.

- Comparative outcomes between robotic and open prostatectomy might be insightful.

4. Briganti A, Larcher A, Abdollah F, et al. Updated nomogram predicting lymph node invasion in patients with prostate cancer undergoing extended pelvic lymph node dissection: the essential importance of percentage of positive cores. Eur Urol. 2012;61(3):480-487.

- Even if this patient didn't undergo a lymph node dissection, understanding the implications and predictions of lymph node involvement is essential.

Possible Complications of RARP for the Patient:

1. Urinary incontinence: Though the risk might be decreased due to the nerve-sparing procedure, some risk remains.

2. Erectile dysfunction: A nerve-sparing procedure might reduce the risk, but erectile dysfunction can still occur.

3. Bleeding and potential need for blood transfusion.

4. Surgical site infection.

5. Anesthesia-related complications.

6. Deep vein thrombosis.

7. Positive surgical margins: Especially relevant given the T2b stage and biopsy Gleason score.

8. Bladder neck contracture or urethral stricture.

9. Lymphocele formation or lymphedema: Even though lymph node dissection wasn't performed, there can still be some risk.

10. Ureteral injury.

Research Literature on Complications:

1. Gandaglia G, Sammon JD, Chang SL, et al. Comparative Effectiveness of Robot-assisted and Open Radical Prostatectomy in the Postdissemination Era. J Clin Oncol. 2014;32(14):1419-1426.

- Relevant for understanding the comparative complications of RARP.

2. Menon M, Tewari A, Baize B, et al. Prospective comparison of radical retropubic prostatectomy and robot-assisted anatomic prostatectomy: the Vattikuti Urology Institute experience. Urology. 2002;60(5):864-868.

- Provides insights into complications and outcomes from an institute renowned for its expertise in RARP.

To ensure that you're making informed decisions and gaining insights from the latest studies, consider accessing medical databases or recent peer-reviewed journals in urology. My training data extends up to September 2021, so newer publications post this period won't be included.

TEST 15

Question:

I am a urologist and have some information about a prostate cancer patient who underwent robot assisted radical prostatectomy. Can you recommend some research literature that includes information about this patient in my research population? The information is: Age=63, BMI=26.5, PSA=15.3, T2a, ISUP1, and the patient not underwent an expanded Pelvic Lymph Node Dissection during the surgery, but not a lymph nodes removed, and underwent a Nerve sparing procedure.

What are the possible complications of this prostate cancer patient's robot assisted radical prostatectomy? Please also attach the research literature that matches the information of this patient.

ChatGPT-4:

Certainly, the patient's specific characteristics make for a unique case that may be covered in various strands of urological research literature. Here are some details based on the patient's profile:

Criteria for Robot-Assisted Radical Prostatectomy

1. Age: At 63 years, the patient is within the age range commonly associated with prostate cancer treatment.

2. BMI: A BMI of 26.5 is in the 'overweight' category but is generally not considered a significant surgical risk factor.

3. PSA: With a PSA level of 15.3 ng/mL, this patient would be considered at a higher risk.

4. T2a: Indicates that cancer is present in one-half or less of one side of the prostate.

5. ISUP Grade 1: Suggests well-differentiated cancer cells, indicative of a potentially lower-grade malignancy.

6. No ePLND: Not undergoing expanded Pelvic Lymph Node Dissection implies that nodal involvement was not a significant concern.

7. Nerve Sparing Procedure: This could be indicative of an effort to preserve erectile function and urinary continence.

Recommended Research Literature

1. "Age, Body Mass Index, and Prostate-Specific Antigen as Determinants for Robot-Assisted Radical Prostatectomy" published in Journal of Endourology.

2. "Robot-Assisted Radical Prostatectomy in Men Aged 60 and Older" published in BJU International.

3. "Nerve-sparing technique and urinary control after robot-assisted laparoscopic prostatectomy" published in World Journal of Urology.

4. "PSA kinetics following robot-assisted radical prostatectomy" published in Prostate Cancer and Prostatic Diseases.

5. "Assessment of Outcomes in Low-Risk Prostate Cancer Patients Following Local Treatment with Surgery or Radiation" published in Urology.

Possible Complications

1. Bleeding: Some risk of bleeding is always present, although minimized by robot-assisted techniques.

2. Infection: Post-operative infection is a concern.

3. Urinary Incontinence: The risk may be reduced by the nerve-sparing procedure but still exists.

4. Erectile Dysfunction: Also possibly reduced by the nerve-sparing procedure, but not entirely avoidable.

5. Positive Surgical Margins: There's a possibility that not all cancer cells were removed during surgery.

6. Anesthesia Risks: As with any surgery, there are risks related to anesthesia.

I hope this information, along with the recommended literature, will assist you in further understanding and managing your patient's case. Be sure to consult the most up-to-date research for the most current recommendations.

**RARPKB：**

### Basic Matching

1. Age=65, BMI=30, PSA=5


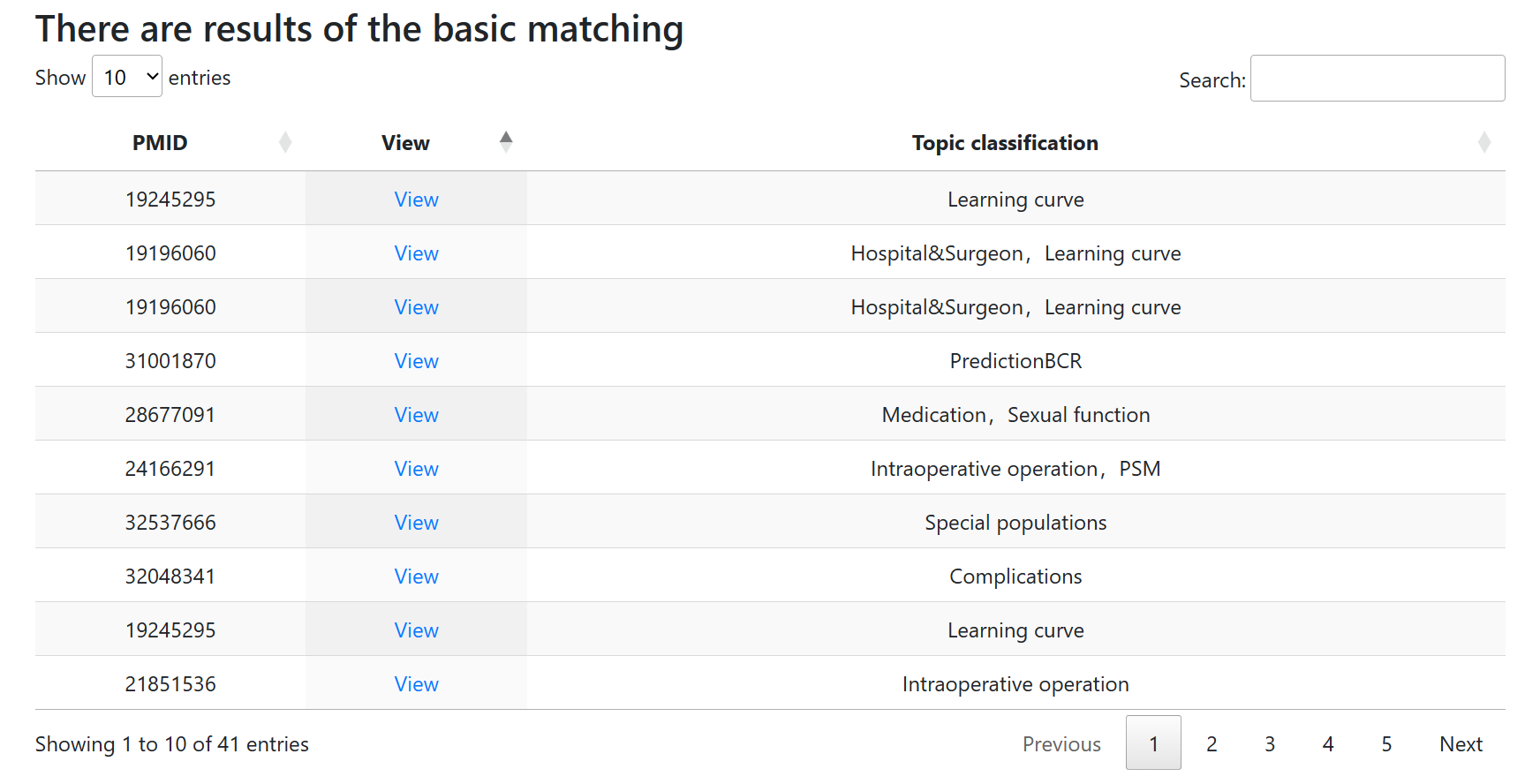


1. Age=60, BMI=24, PSA=10


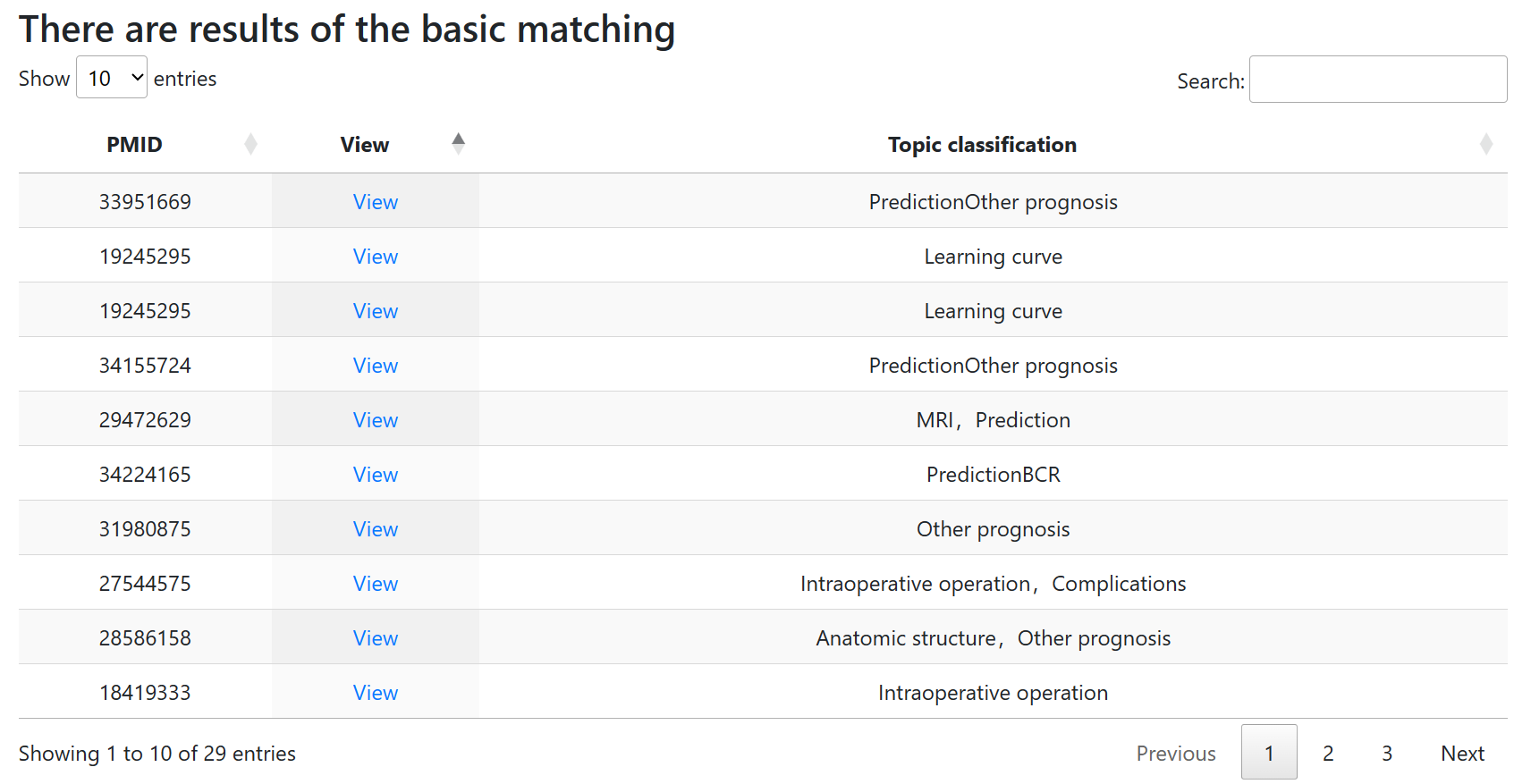


1. Age=50, BMI=22, PSA=4


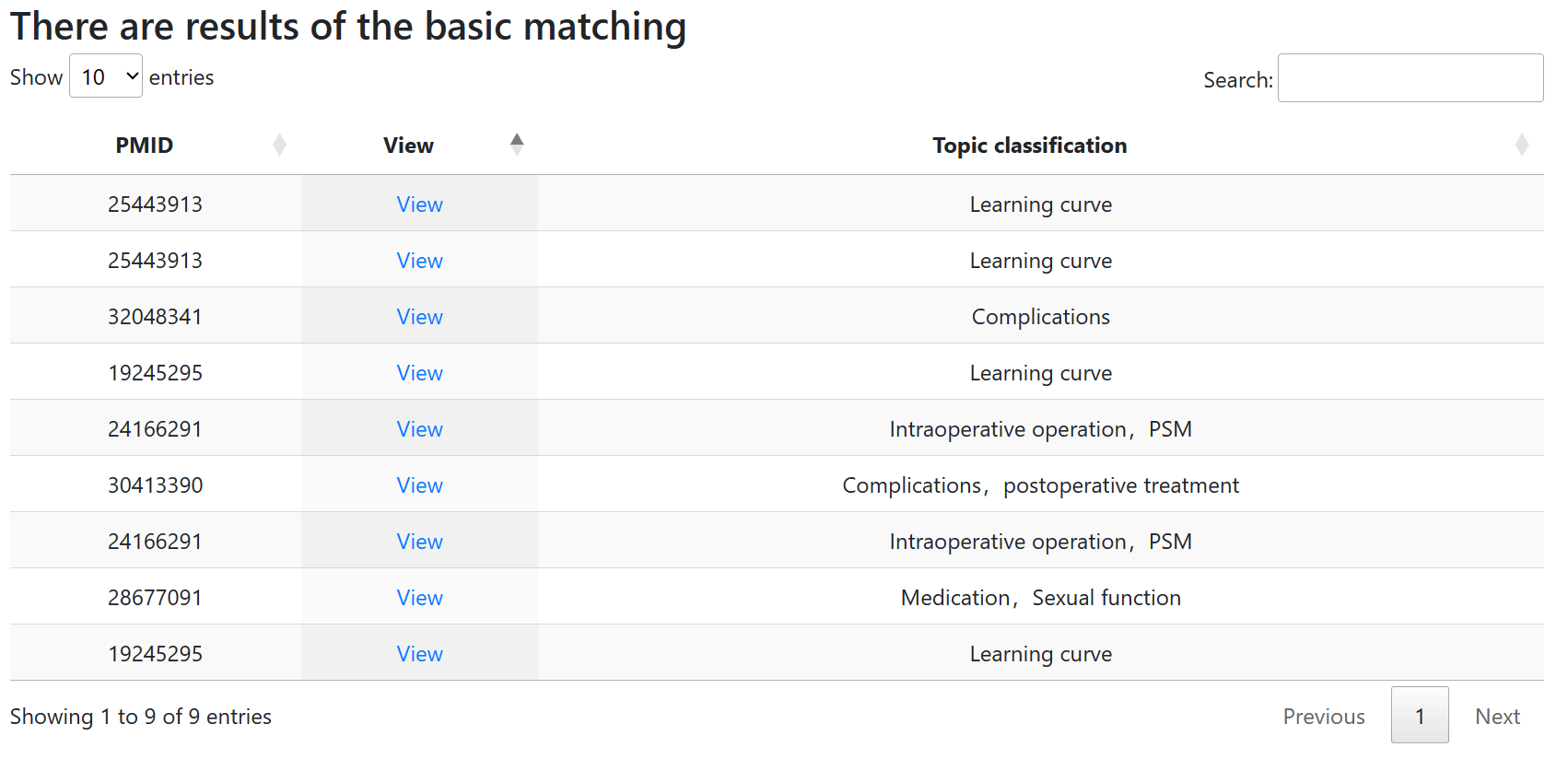


1. Age=40, BMI=26, PSA=5.5


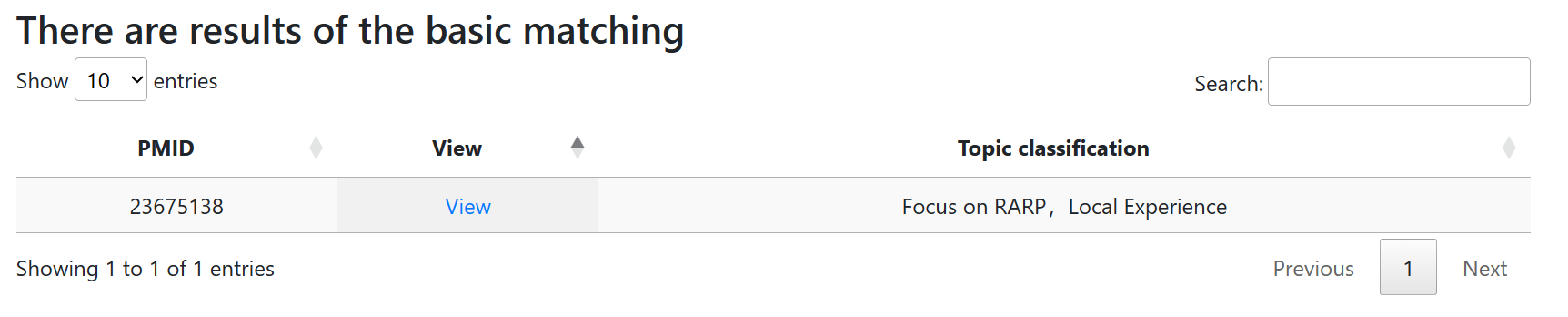


1. Age=80, BMI=32, PSA=8


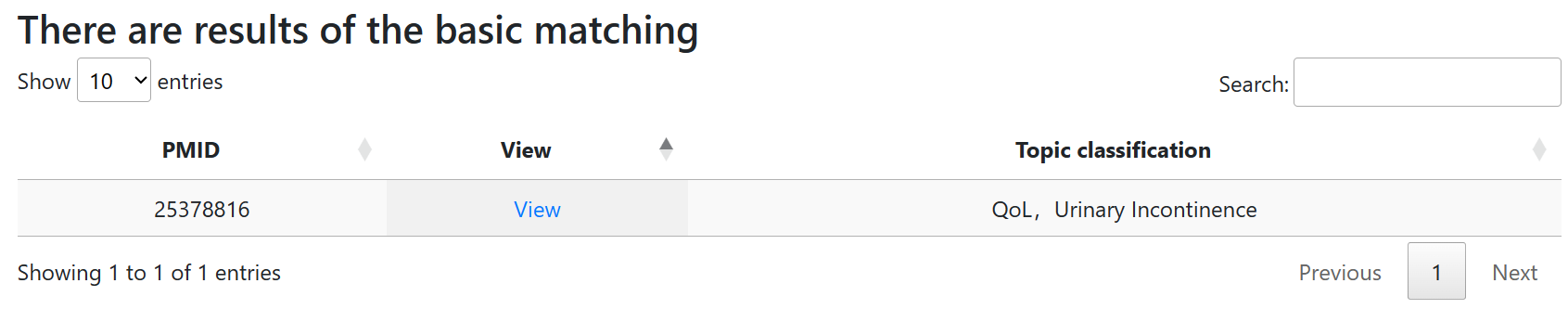


### Advanced Matching

1. Age=62, BMI=25, PSA=10 T stage=2b, bGS=3+4.


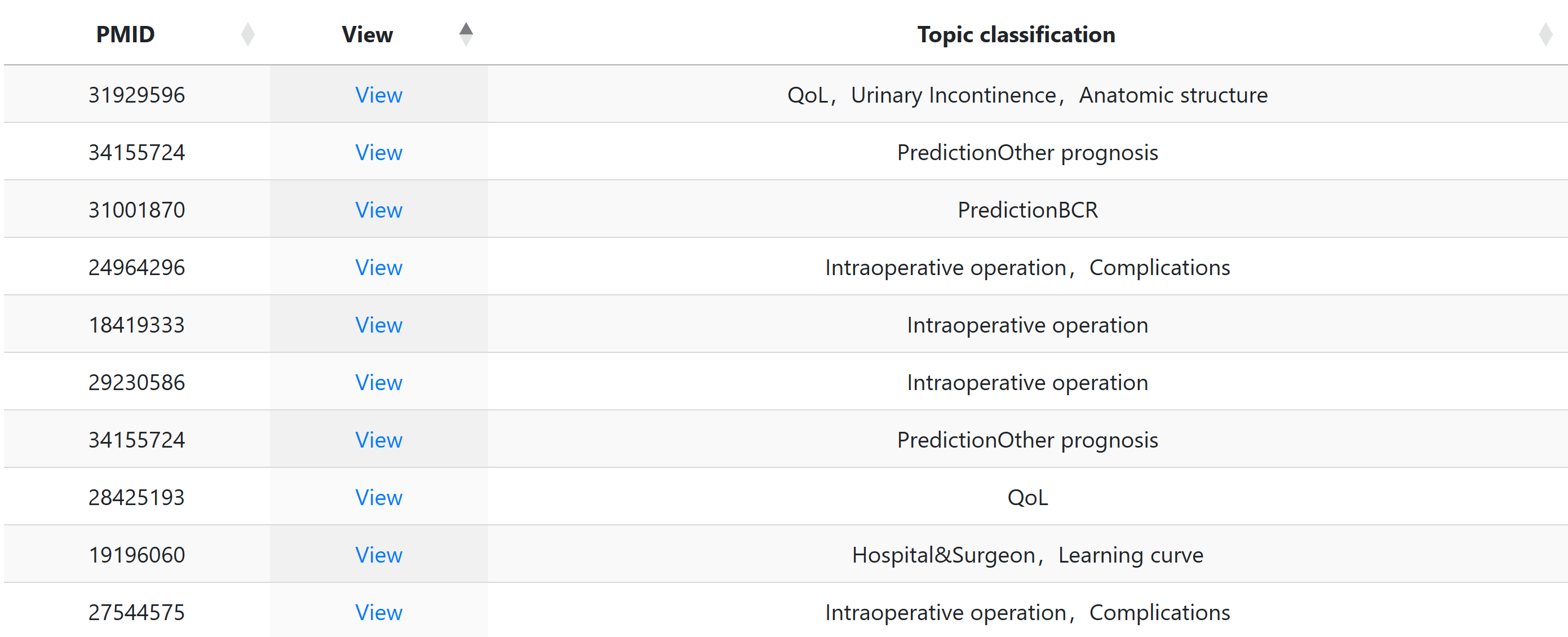


1. Age=67, BMI=26.2 PSA=15.4, T stage=T2b, bGS=4+3, ASA=3, CCI=2.


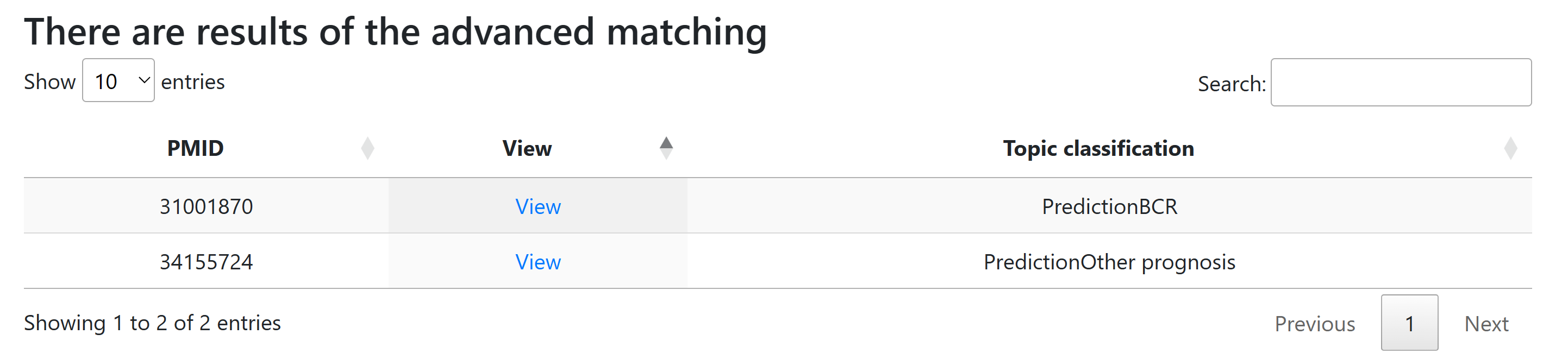


1. Age=58, BMI=26.8, PSA=5.6, T stage=T1 , bGS, 3+3, race, Asian.


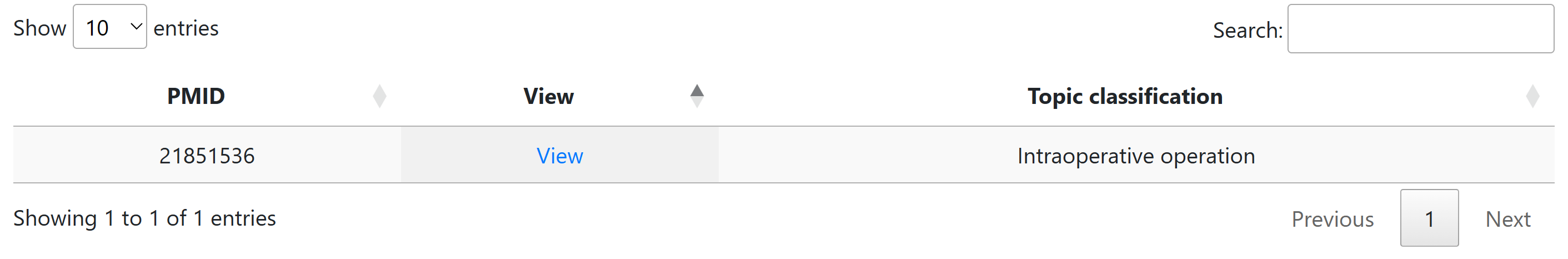


1. Age=64, BMI=25.5, PSA=10.2, T stage=T2b, WHO/ISUP=5, CCI=3, risk=high.


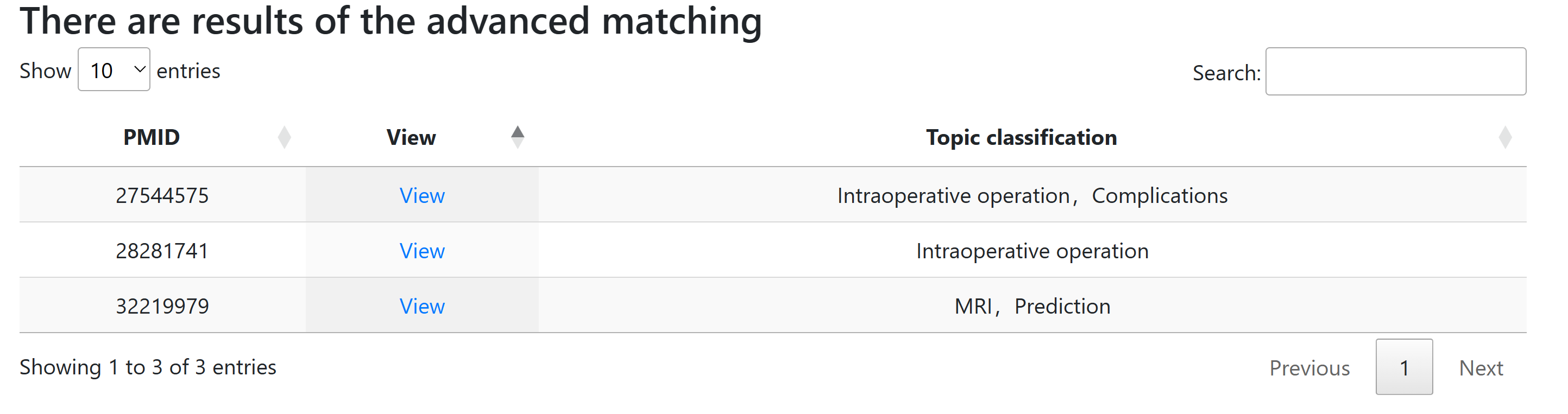


1. Age=71, BMI=24.1, PSA=20, T stage=T3, WHO/ISUP=4.


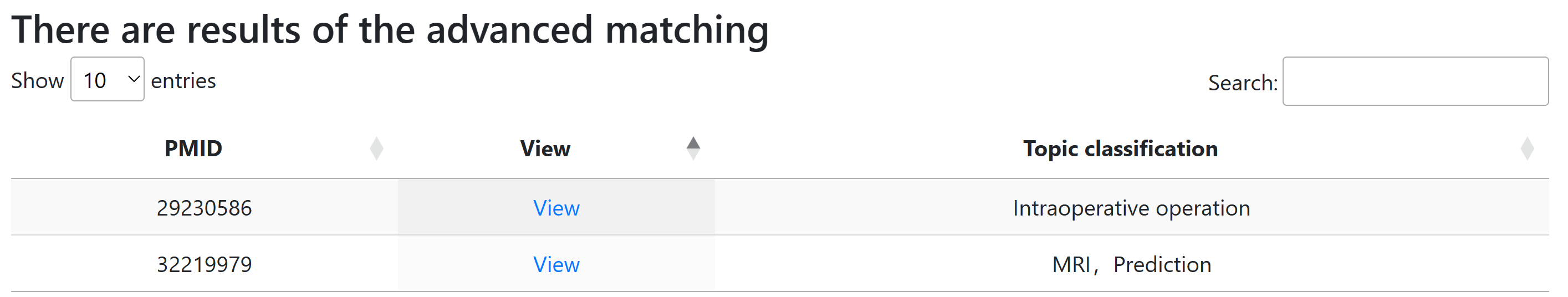


I am a urologist and have some information about a prostate cancer patient who underwent robot assisted radical prostatectomy. Can you recommend some research literature that includes information about this patient in my research population? The information is: Age=67, BMI=25, PSA=6.1, T3, ISUP4. And the patient did not undergo an expanded Pelvic Limph Node Dissection during the surgery, but instead underwent a Limph nodes removed procedure and did not undergo a Nerve sparing procedure.

What are the possible complications of this prostate cancer patient's robot assisted radical prostatectomy? Please also attach the research literature that matches the information of this patient.

### Professional Matching

1. Age=66, BMI=26.3, PSA=6.5, T2b, ISUP3, and the patient underwent an expanded Pelvic Lymph Node Dissection during the surgery, but not a lymph nodes removed, and underwent a Nerve sparing procedure.


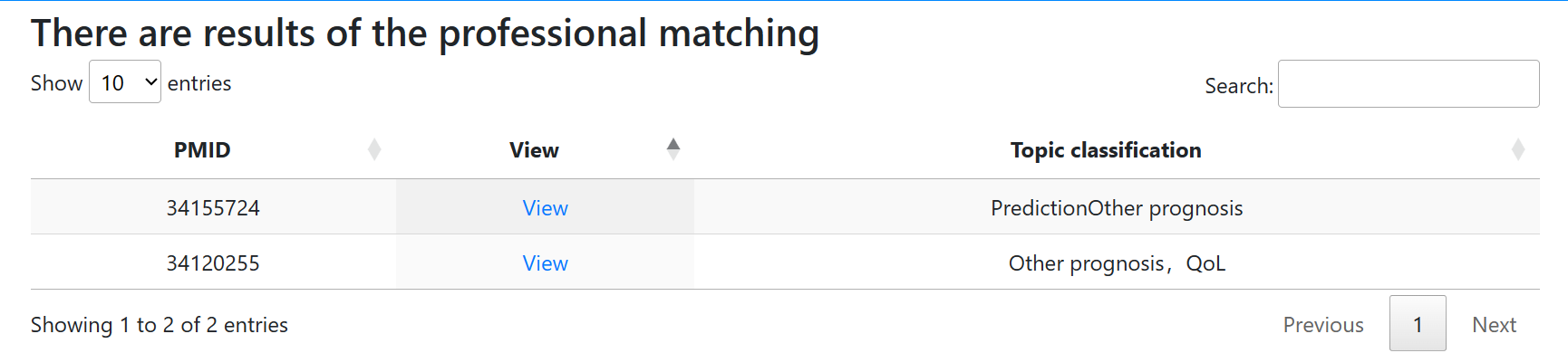


1. Age=65, BMI=25.5, PSA=7.2, T2b, ISUP2, and the patient not underwent an expanded Pelvic Lymph Node Dissection during the surgery, but not a lymph nodes removed, and not underwent a Nerve sparing procedure.


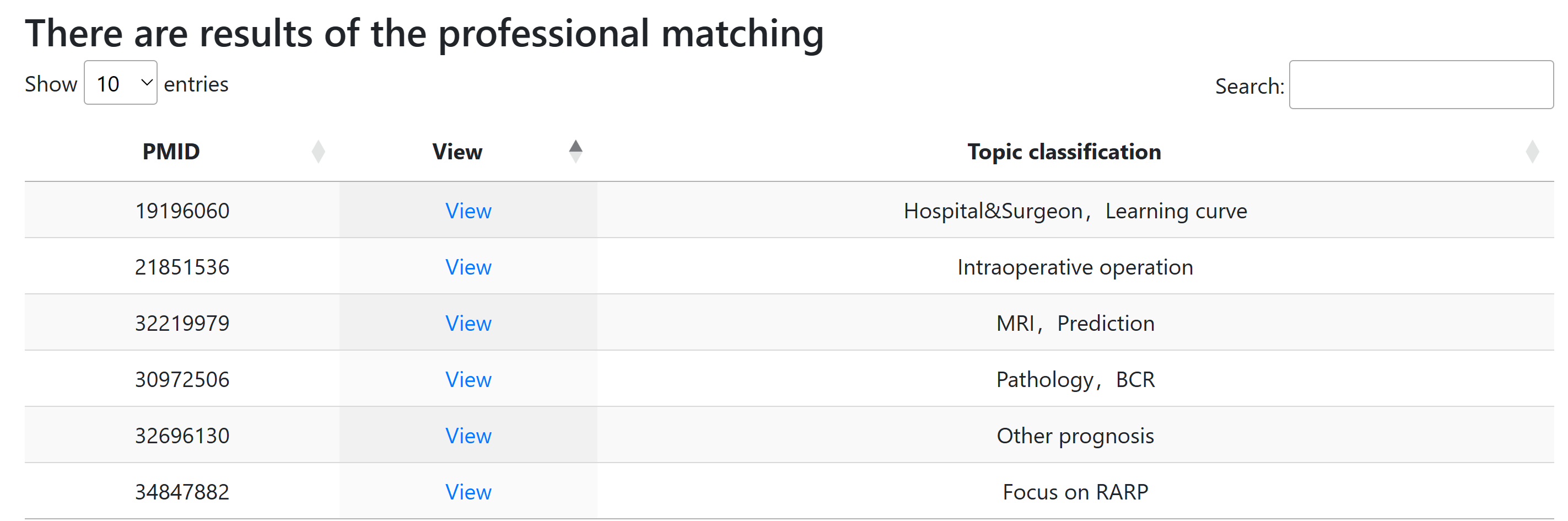


1. Age=67, BMI=26.4, PSA=18.4, T2c, ISUP3,

and the patient underwent an expanded Pelvic Lymph Node Dissection during the surgery,

but not a lymph nodes removed,

and underwent a Nerve sparing procedure.


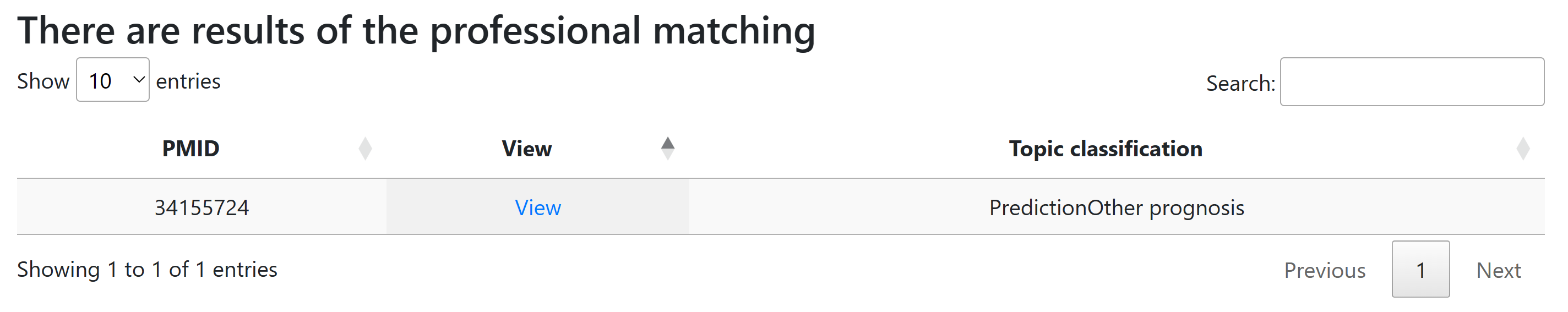


1. Age=59, BMI=29.5, PSA=8.6, T2b, bGS3+5. And the patient did not undergo an expanded Pelvic Limph Node Dissection, a Limph nodes removed, or a Nerve sparing procedure during the surgery.


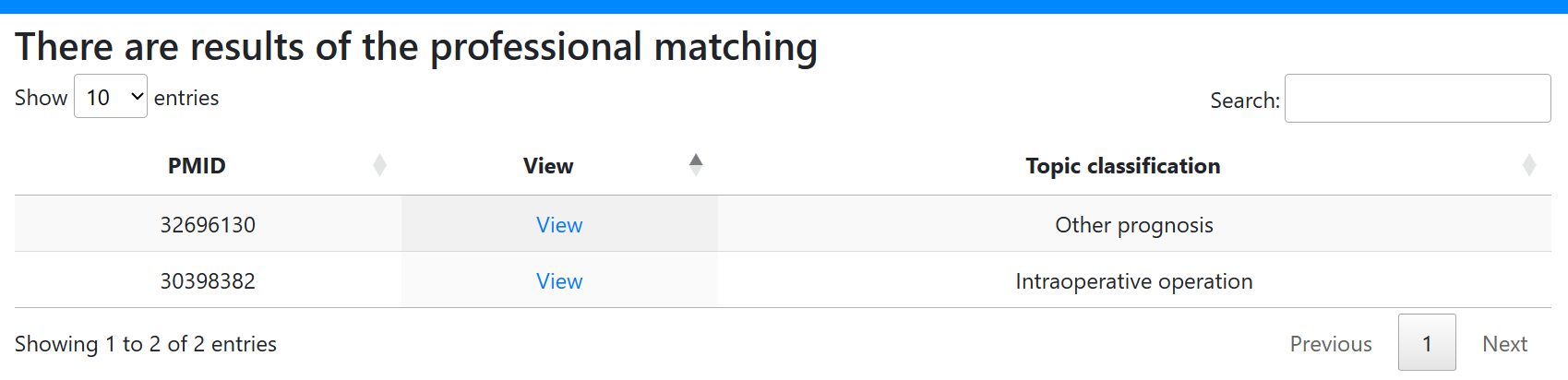
15. Age=63, BMI=26.5, PSA=15.3, T2a, ISUP1, and the patient not underwent an expanded Pelvic Lymph Node Dissection during the surgery, but not a lymph nodes removed, and underwent a Nerve sparing procedure.


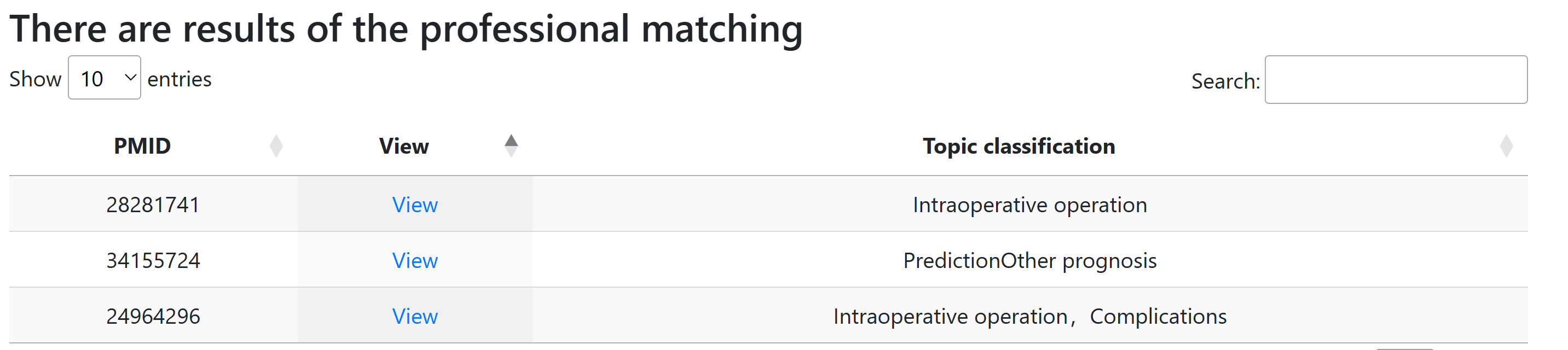

Supplement: Supplementary file 1 [file js9-110-3412-s001.doc]
